# Correlates of protection against symptomatic and asymptomatic SARS-CoV-2 infection

**DOI:** 10.1038/s41591-021-01540-1

**Published:** 2021-09-29

**Authors:** Shuo Feng, Daniel J. Phillips, Thomas White, Homesh Sayal, Parvinder K. Aley, Sagida Bibi, Christina Dold, Michelle Fuskova, Sarah C. Gilbert, Ian Hirsch, Holly E. Humphries, Brett Jepson, Elizabeth J. Kelly, Emma Plested, Kathryn Shoemaker, Kelly M. Thomas, Johan Vekemans, Tonya L. Villafana, Teresa Lambe, Andrew J. Pollard, Merryn Voysey, the Oxford COVID Vaccine Trial Group, Syed Adlou, Lauren Allen, Brian Angus, Rachel Anslow, Marie-Claude Asselin, Natalie Baker, Philip Baker, Thomas Barlow, Amy Beveridge, Kevin R. Bewley, Phillip Brown, Emily Brunt, Karen R. Buttigieg, Susana Camara, Sue Charlton, Emily Chiplin, Paola Cicconi, Elizabeth A. Clutterbuck, Andrea M. Collins, Naomi S. Coombes, Sue Ann Costa Clemens, Melanie Davison, Tesfaye Demissie, Tanya Dinesh, Alexander D. Douglas, Christopher J. A. Duncan, Katherine R. W. Emary, Katie J. Ewer, Sally Felle, Daniela M. Ferreira, Adam Finn, Pedro M. Folegatti, Ross Fothergill, Sara Fraser, Harriet Garlant, Laura Gatcombe, Kerry J. Godwin, Anna L. Goodman, Christopher A. Green, Bassam Hallis, Thomas C. Hart, Paul T. Heath, Helen Hill, Adrian V. S. Hill, Daniel Jenkin, Mwila Kasanyinga, Simon Kerridge, Chanice Knight, Stephanie Leung, Vincenzo Libri, Patrick J. Lillie, Spyridoula Marinou, Joanna McGlashan, Alastair C. McGregor, Lorna McInroy, Angela M. Minassian, Yama F. Mujadidi, Elizabeth J. Penn, Christos J. Petropoulos, Katrina M. Pollock, Pamela C. Proud, Samuel Provstgaard-Morys, Durga Rajapaska, Maheshi N. Ramasamy, Katherine Sanders, Imam Shaik, Nisha Singh, Andrew Smith, Matthew D. Snape, Rinn Song, Sonu Shrestha, Rebecca K. Sutherland, Emma C. Thomson, David P. J. Turner, Alice Webb-Bridges, Terri Wrin, Christopher J. Williams

**Affiliations:** 1 Oxford Vaccine Group, Department of Paediatrics, University of Oxford, Oxford, UK; 2 Late-stage development, Respiratory and Immunology (R&I), BioPharmaceuticals R&D, AstraZeneca, Cambridge, UK; 3 The Jenner Institute, Nuffield Department of Medicine, University of Oxford, Oxford, UK; 4 National Infection Service, Public Health England, Salisbury, UK; 5 Late-stage development, Respiratory and Immunology (R&I), BioPharmaceuticals R&D, AstraZeneca, Gaithersburg, MD, USA; 6 Cytel Inc., Cambridge, MA, USA; 7 Microbial Sciences, BioPharmaceuticals R&D, AstraZeneca, Gaithersburg, MD, USA; 8 Late-stage development Respiratory and Immunology (R&I), BioPharmaceuticals R&D, AstraZeneca, Gothenburg, Sweden; 9 Chinese Academy of Medical Science (CAMS) Oxford Institute (COI), University of Oxford, Oxford, UK; 10 NIHR Oxford Biomedical Centre, Oxford, UK; 11 Department of Clinical Sciences, Liverpool School of Tropical Medicine, Liverpool, UK; 12 Liverpool University Hospitals NHS Foundation Trust, Liverpool, UK; 13 Institute of Global Health, University of Siena, Siena, Italy; 14 Department of Infection and Tropical Medicine, Newcastle upon Tyne Hospitals NHS Foundation Trust, Newcastle, UK; 15 Translational and Clinical Research Institute, Immunity and Inflammation Theme, Newcastle University, Newcastle, UK; 16 School of Population Health Sciences, University of Bristol, Bristol, UK; 17 University Hospitals Bristol and Weston NHS Foundation Trust, Bristol, UK; 18 Department of Infectious Diseases, Guy’s and St Thomas’ NHS Foundation Trust, St Thomas’ Hospital, London, UK; 19 MRC Clinical Trials Unit at University College London, London, UK; 20 NIHR/Wellcome Trust Clinical Research Facility, University Hospitals Birmingham NHS Foundation Trust, Birmingham, UK; 21 Institute of Microbiology & Infection, University of Birmingham, Birmingham, UK; 22 St George’s Vaccine Institute, St George’s, University of London, London, UK; 23 NIHR UCLH Clinical Research Facility, London, UK; 24 NIHR UCLH Biomedical Research Centre, London, UK; 25 Hull University Teaching Hospitals NHS Trust and Hull York Medical School, Hull, UK; 26 London North West University Healthcare NHS Trust, London, UK; 27 Department of Medicine, Imperial College London, London, UK; 28 Labcorp-Monogram Biosciences, South San Francisco, CA USA; 29 NIHR Imperial Clinical Research Facility and NIHR Imperial Biomedical Research Centre, London, UK; 30 College of Medical, Veterinary & Life Sciences, Glasgow Dental Hospital & School, University of Glasgow, Glasgow, UK; 31 Clinical Infection Research Group, Regional Infectious Diseases Unit, NHS Lothian, Edinburgh, UK; 32 MRC - University of Glasgow Centre for Virus Research & Department of Infectious Diseases, Queen Elizabeth University Hospital, Glasgow, UK; 33 University of Nottingham and Nottingham University Hospitals NHS Trust, Nottingham, UK; 34 Aneurin Bevan University Health Board, Newport, UK

**Keywords:** Infectious diseases, Vaccines

## Abstract

The global supply of COVID-19 vaccines remains limited. An understanding of the immune response that is predictive of protection could facilitate rapid licensure of new vaccines. Data from a randomized efficacy trial of the ChAdOx1 nCoV-19 (AZD1222) vaccine in the United Kingdom was analyzed to determine the antibody levels associated with protection against SARS-CoV-2. Binding and neutralizing antibodies at 28 days after the second dose were measured in infected and noninfected vaccine recipients. Higher levels of all immune markers were correlated with a reduced risk of symptomatic infection. A vaccine efficacy of 80% against symptomatic infection with majority Alpha (B.1.1.7) variant of SARS-CoV-2 was achieved with 264 (95% CI: 108, 806) binding antibody units (BAU)/ml: and 506 (95% CI: 135, not computed (beyond data range) (NC)) BAU/ml for anti-spike and anti-RBD antibodies, and 26 (95% CI: NC, NC) international unit (IU)/ml and 247 (95% CI: 101, NC) normalized neutralization titers (NF_50_) for pseudovirus and live-virus neutralization, respectively. Immune markers were not correlated with asymptomatic infections at the 5% significance level. These data can be used to bridge to new populations using validated assays, and allow extrapolation of efficacy estimates to new COVID-19 vaccines.

## Abstract (web summary)

Defined levels of SARS-CoV-2-specific binding and neutralizing antibodies elicited by the COVID-19 vaccine ChAdOx1 nCoV-19 are identified as correlates of protection against symptomatic infection.

## Main

Within 17 months of the identification of SARS-CoV-2 in Wuhan, China, in response to the pandemic, 6 COVID-19 vaccines were recommended for use by the World Health Organization (WHO) as of 16 June 2021 (ref. ^1^). Vaccine efficacies (VE) ranging from 50% to 95% against symptomatic COVID-19 infections have been reported, using varying endpoint definitions^2–7^. Real-world evidence from vaccine-rollout programs has shown that COVID-19 vaccines are highly effective against severe disease, hospitalization, and death, and reduce both asymptomatic infection and within household transmission^8–13^.

The global supply of COVID-19 vaccines remains limited despite intense production efforts. Authorization of new vaccines could help meet demand. As more countries implement vaccine programs, it will become increasingly difficult to conduct clinical efficacy studies of new vaccines. Understanding the relationship between immune responses to vaccines and protection against clinical outcomes is urgently needed to speed vaccine development. Knowledge of immune measures that are statistically associated with protection against disease (‘correlates of protection’) may allow new vaccines to be authorized for use on the basis of immunogenicity and safety data alone, when large efficacy trials are not feasible. In addition, understanding the immune response allows for comparison of vaccines across cohorts of people who differ by age, race, ethnicity, or other factors.

Both binding and neutralizing antibodies are thought to be potential correlates of protection against COVID-19 and are correlated with each other^3,14–16^. Previous human challenge studies of seasonal coronaviruses reported high levels of baseline neutralizing antibodies in uninfected or asymptomatic people^17^. However, protection from infection with seasonal coronaviruses is not long-lasting^17,18^.

Early evidence from a fishery-vessel outbreak of SARS-CoV-2 suggested that higher levels of pre-existing neutralizing antibodies were potential correlates of protection^18,19^. A longitudinal cohort study of healthcare workers highlighted the association between baseline anti-spike and anti-nucleocapsid immunoglobulin G (IgG) and decreased risk of SARS-CoV-2 infection in the following 6 months^19,20^.

Evidence that antibodies may play a role in mediating protection against overt disease has come from vaccination and challenge studies in animals. Both neutralizing antibody titers and Fc-dependent functional antibody responses correlate with protection induced by DNA and adenoviral vector vaccines in rhesus macaques (*Macaca mulatta*)^21,22^. Additionally, higher doses of passively transferred monoclonal antibodies were more protective than were lower doses in golden hamsters (*Mesocricetus auratus*) and rhesus macaques challenged with the SARS-CoV-2 virus containing D614 in its spike protein^23^.

A meta-analysis modeling the relationship between VE reported from phase 3 vaccine clinical trials and neutralization titers in convalescent patients showed a significant association at the study level between VE and neutralizing antibody levels^24^. Nevertheless, no study to date has defined a correlate of protection against SARS-CoV-2 infection or disease that can be used by regulators and vaccine developers.

The ChAdOx1 nCoV-19 vaccine (AZD1222) is a chimpanzee adenoviral vector vaccine with full-length SARS-CoV-2 spike insert which was developed at the University of Oxford and is in widespread global use and produced by AstraZeneca and their manufacturing partners. Using data from the United Kingdom and Brazil, we previously estimated an overall VE of 66.7% (95% confidence interval (CI): 57.4 to 74.0) against symptomatic infection and 27.3% (95% CI: −17.2 to 54.9) against asymptomatic infection^2,3^. We previously showed that estimates of VE against symptomatic COVID-19 infection were higher in subgroups with higher pseudovirus neutralization antibody titers, or higher anti-spike IgG levels, in vaccine clinical trials of ChAdOx1 nCoV-19 in adults using summarized data^3^. Here, we report the relationship between a continuous measure of the humoral immune responses to vaccination and protection afforded by this vaccine, which may facilitate further vaccine development. Specifically, we used individual data from the United Kingdom and identified the thresholds for four immune markers associated with protection against symptomatic infection. The WHO international standard units are reported for all assays, to allow comparisons across studies and platforms.

## Results

Using the COV002 data from the United Kingdom, we assessed the correlation between immune markers at 28 days post the second dose (post-boost + 28 days, PB28) of ChAdOx1 nCoV-19 and symptomatic and asymptomatic infections. Participants were reminded weekly to contact their study site if they experienced any of the primary symptoms of COVID-19 (fever ≥ 37.8 °C; cough; shortness of breath; anosmia or ageusia) and were assessed in clinic with a nose and throat swab taken for nucleic acid amplification testing (NAAT). Additionally, participants were asked to complete a nose and throat swab at home each week, which was used to detect asymptomatic infections. Nucleic acid amplification test positive (NAAT+) participants who had symptoms other than the main five COVID-19 symptoms were categorized as nonprimary symptomatic and were not included in correlates analysis.

**Extended Data Fig. 1:**
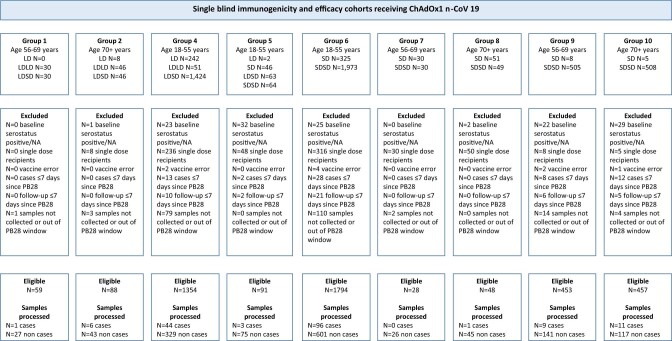
Participant flow chart showing inclusion in correlates models. Eligible participants comprised the Correlates Population and those with samples processed comprised the Correlates Cohort.

Table 1 summarizes baseline characteristics for the defined correlates population, control population, and correlates cohort by status (cases and noncases). Extended Data Fig. 1 summarizes the exclusions for each study group. Participants were followed for a median of 88 and 85 days, counting from 7 days after the PB28 visit, among correlates and control populations, respectively. The follow-up time was censored at the earliest timing of infection, withdrawal, or unblinding, or the cut-off date 28 February 2021. Among 4,372 participants in the correlates population, there were a total of 174 breakthrough NAAT+ cases of SARS-CoV-2 infection. Data were available for at least one of four assay readouts (anti-spike IgG, anti-receptor binding domain (RBD) IgG, pseudovirus neutralization assay, and live-virus neutralization) for 171 out of 174 (98.3%) cases and 1,404 out of 4,195 (33.5%) noncases. Data were available for anti-spike and anti-RBD IgG from 1,318 PB28 samples (163 cases and 1,155 noncases, Supplementary Table 2). A smaller set of data was available for analysis for pseudovirus neutralization titers (149 cases, 828 noncases) and for live-virus neutralization (110 cases and 412 noncases) (Supplementary Table 2). People in the case group were younger, with 84.2% being aged 18–55 years, compared with 71.6% of the noncase group, and were more likely to be healthcare workers (62.0% were healthcare workers compared with 57.5% of the noncase group, Table 1). In our baseline exposure model developed among the MenACWY control group, younger age and being a healthcare worker facing more than one patient with COVID-19 per day were associated with a higher risk of being NAAT+. Other variables were not significant (see model output in Supplementary Table 3). The distribution of baseline risk was similar for cases and noncases (Table 1).

**Table 1:** Baseline characteristics of correlates population, control population, and cases and noncases among correlates cohort

	ChAdOx1 nCoV-19 correlates population (*n* = 4,372)	MenACWY control population (*n* = 4,194)	ChAdOx1 nCoV-19 correlates cohort^a^	
			Cases (*n* = 171)	Noncases (*n* = 1,404)
Age group				
18–55 years	3,240 (74.1%)	3,229 (77%)	144 (84.2%)	1,005 (71.6%)
56–69 years	542 (12.4%)	482 (11.5%)	10 (5.8%)	194 (13.8%)
≥70 years	590 (13.5%)	483 (11.5%)	17 (9.9%)	205 (14.6%)
Sex (Female)	2,533 (57.9%)	2,526 (60.2%)	102 (59.6%)	780 (55.6%)
Ethnicity				
White	4,036 (92.3%)	3,914 (93.3%)	160 (93.6%)	1,293 (92.1%)
Asian	220 (5.0%)	184 (4.4%)	8 (4.7%)	71 (5.1%)
Black	21 (0.5%)	15 (0.4%)	1 (0.6%)	10 (0.7%)
Other^b^	95 (2.2%)	81 (1.9%)	2 (1.2%)	30 (2.1%)
BMI (mean (s.d.))	26.4 (5)	26.5 (5.2)	27 (5.2)	26.5 (5.1)
BMI < 30	3,519 (80.5%)	3,347 (79.8%)	130 (76.0%)	1,124 (80.1%)
BMI ≥ 30	852 (19.5%)	846 (20.2%)	41 (24.0%)	280 (19.9%)
Comorbidities	1,088 (24.9%)	1,032 (24.6%)	44 (25.7%)	360 (25.6%)
Respiratory disease	547 (12.5%)	537 (12.8%)	20 (11.7%)	178 (12.7%)
Cardiovascular disease	572 (13.1%)	514 (12.3%)	24 (14.0%)	192 (13.7%)
Diabetes	99 (2.3%)	85 (2%)	3 (1.8%)	36 (2.6%)
Healthcare worker status				
Nonhealthcare worker	1,652 (37.8%)	1,456 (34.7%)	65 (38.0%)	597 (42.5%)
Healthcare worker facing no more than one patient with COVID-19 per day	1,904 (43.6%)	1,938 (46.2%)	74 (43.3%)	587 (41.8%)
Healthcare worker facing at least one patient with COVID-19 per day	816 (18.7%)	800 (19.1%)	32 (18.7%)	220 (15.7%)
Baseline risk probabilities^c^				
Mean (s.d.)	0.0786 (0.0303)	0.0794 (0.0296)	0.0824 (0.0283)	0.0774 (0.0306)
Dosage schedule				
LD/LD	125 (2.9%)	69 (1.6%)	7 (4.1%)	114 (8.1%)
LD/SD	1,420 (32.5%)	1,361 (32.5%)	46 (26.9%)	320 (22.8%)
SD/SD	2,827 (64.7%)	2,764 (65.9%)	118 (69%)	970 (69.1%)
Prime-boost interval				
<6 weeks	1,078 (24.7%)	931 (22.2%)	28 (16.4%)	456 (32.5%)
6–8 weeks	538 (12.3%)	478 (11.4%)	43 (25.1%)	197 (14%)
9–11 weeks	1,158 (26.5%)	1,236 (29.5%)	42 (24.6%)	398 (28.3%)
≥12 weeks	1,598 (36.6%)	1,549 (36.9%)	58 (33.9%)	353 (25.1%)
Length of follow-up (days) from 7 days post PB28 until infection occurred or Feb 28 2021 (median (IQR))	88 (64, 113)	85 (62, 108)	53 (29, 81)	105 (81, 135)
NAAT+ cases	174	333	171	
Symptomatic	55 (31.6%)	196 (58.9%)	54 (31.6%)	
Asymptomatic	99 (56.9%)	112 (33.6%)	97 (56.7%)	
Nonprimary symptomatic	20 (11.5%)	25 (7.5%)	20 (11.7%)	

BMI, body mass index; LD, low dose; SD, standard dose

^a^The correlates cohort is a subset of all eligible participants in the ChAdOx1 nCoV-19 correlates populations who have samples processed for at least one assay.

^b^Options included in ‘Other’ are as follows: ‘Mixed’, ‘Other – Free text’, or ‘prefers not to give’.

^c^The baseline risk exposure score summarizes predicted probability of having NAAT+ outcome from the risk model developed using the MenACWY Control Population.

**Extended Data Fig. 2:**
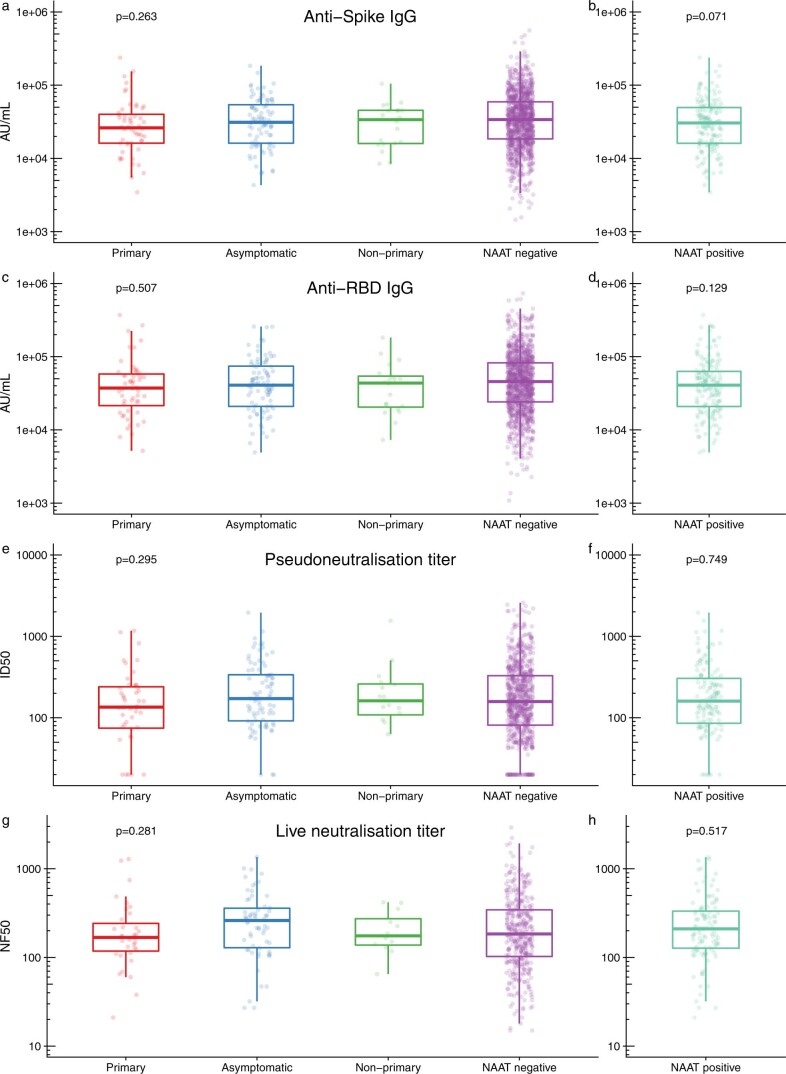
Immune markers measured at day 28 post-second dose, in primary symptomatic, asymptomatic, non-primary cases, NAAT positive cases and NAAT negative non-cases. **a**: N = 1155 NAAT negative, 52 primary, 91 asymptomatic and 20 non-primary, **b**: N = 163 NAAT positive participants’ anti-spike IgG measured at 28 days post boost; **c**: N = 1155 negative, 52 primary, 91 asymptomatic and 20 non-primary, d**: N** = 163 NAAT positive participants’ anti-RBD IgG measured at 28 days post boost; **e**: N = 828 NAAT negative, 47 primary, 86 asymptomatic and 16 non-primary, **f**: N = 149 NAAT positive participants’ pseudovirus neutralisation titre measured at 28 days post boost; **g**: N = 412 negative, 36 primary, 62 asymptomatic and 12 non-primary, **h**: N = 110 NAAT positive participants’ live neutralisation titre measured at 28 days post boost. **a**–**h**: minima: smallest value; maxima: largest value; centre: median value; bounds of box: 25% and 75% quartile value; upper/lower whisker extends from the hinge to the largest/smallest value no further than 1.5 * inter-quartile range from the hinge. IgG: Immunoglobulin G; RBD: receptor binding domain. Primary symptomatic cases: NAAT+ with at least one COVID symptom (cough, fever, shortness of breath, anosmia, aguesia). Asymptomatic cases: NAAT+ on weekly self-swab with no symptoms recorded. Non-primary cases: NAAT+ with only non-primary COVID symptoms (for example nausea, diarrhoea). P-value estimated by one-way ANOVA test comparing between primary, asymptomatic, non-primary cases and NAAT negative non-cases and by two sample t-test comparing between NAAT positive cases and NAAT negative non-cases (two-sided).

**Extended Data Fig. 3:**
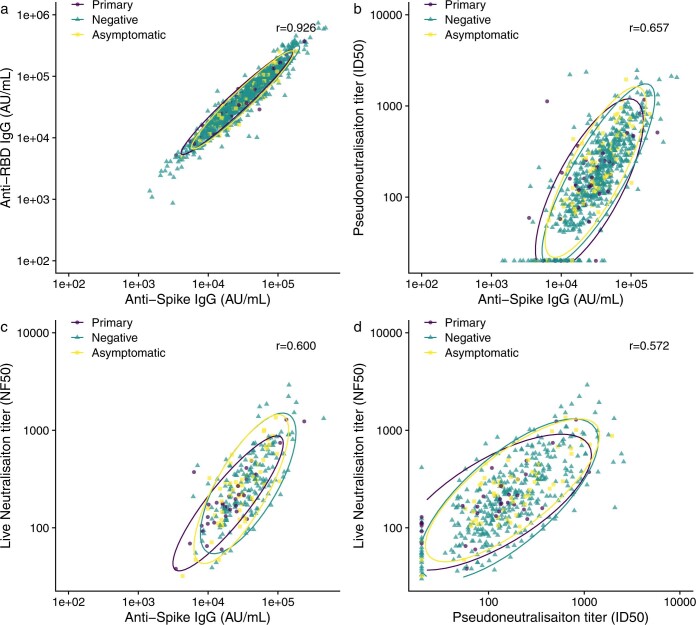
Correlations between a, Anti-SARS-CoV-2 spike and RBD IgG. b, Anti-SARS-CoV-2 Spike IgG and pseudovirus neutralisation titre. c, Anti-SARS-CoV-2 Spike IgG and live virus neutralisation titre. d, pseudovirus neutralisation titres and live virus neutralisation titres. 95% confidence ellipses assuming a t-distribution are shown for each outcome (primary symptomatic cases, asymptomatic cases and negative controls). Pearson correlation coefficients shown as r values using all available data. Primary symptomatic cases: NAAT+ with at least one COVID symptom (cough, fever, shortness of breath, anosmia, aguesia). Asymptomatic cases: NAAT+ on weekly self-swab with no symptoms recorded.

Antibody levels at PB28 in cases and noncases across four immune markers are shown in Extended Data Fig. 2. Anti-spike IgG and anti-RBD IgG were highly correlated with each other (Pearson correlation coefficient *r* = 0.926), while the correlation between pseudovirus neutralization titer and normalized live-virus neutralization titer (NF_50_) was moderate (*r* = 0.572). Anti-spike IgG values were also correlated with pseudovirus neutralization titers (*r* = 0.657) and normalized live-virus neutralization titers (NF_50_) (*r* = 0.600) (Extended Data Fig. 3). Non-normalized live-virus neutralization titers (ND_50_) were less highly correlated with anti-spike IgG (*r* = 0.411) and pseudovirus neutralization titers (*r* = 0.305).

**Fig. 1:**
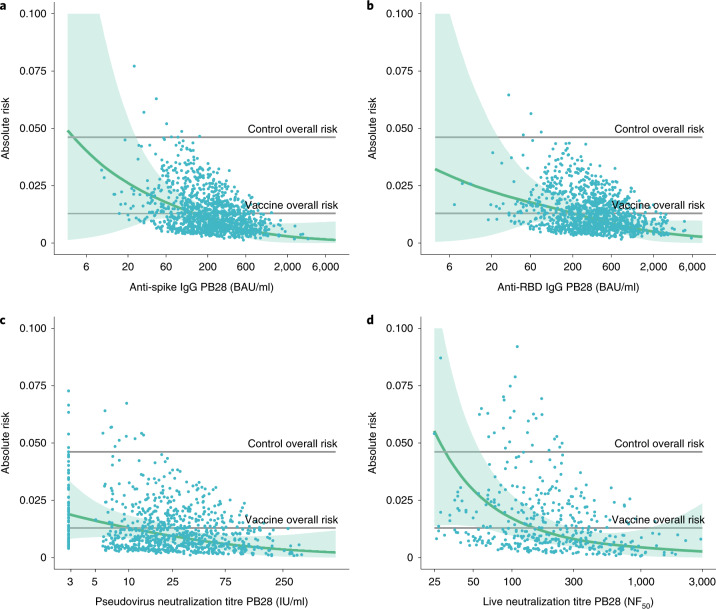
Predicted absolute risk of primary symptomatic COVID-19 as a function of immune markers measured at PB28 by generalized additive regression. **a**–**d**, Predicted absolute risk of primary symptomatic COVID-19 as a function of anti-spike IgG measured at PB28 (52 cases, 1,155 noncases included in the analysis) (**a**), anti-RBD IgG measured at PB28 (52 cases, 1,155 noncases included in the analysis) (**b**), pseudovirus neutralization antibody titers at PB28 (47 cases, 828 noncases included in the analysis (**c**), and live-virus neutralization antibody titers PB28 (36 cases, 412 noncases included in the analysis) (**d**). Gray horizontal lines show the overall risk of primary symptomatic COVID-19 in the control group (MenACWY) and vaccine groups (ChAdOx1 nCoV-19). Blue dots show the absolute risk predicted from the model across the range of antibody values included in the analysis, adjusting for baseline exposure risk to SARS-CoV-2 infection. Green shaded areas show the CI around the predicted mean probability (green line).

**Fig. 2:**
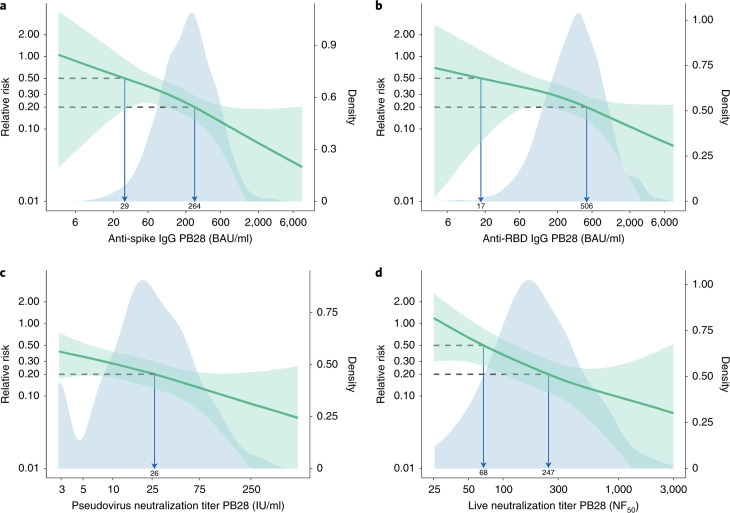
Relative risk of primary symptomatic COVID-19 among vaccine recipients compared with MenACWY control arm participants as a function of immune markers measured at PB28. **a**, Anti-spike IgG measured at PB28 (52 cases, 1,155 noncases included in the analysis). **b**, Anti-RBD IgG measured at PB28 (52 cases, 1,155 noncases included in the analysis). **c**, Pseudovirus neutralization antibody titers at PB28 (47 cases, 828 noncases included in the analysis). **d**, Live-virus neutralization antibody titers at PB28 (36 cases, 412 noncases included in the analysis). Blue shaded areas represent the immune marker density distribution. Green lines show the relative risk of infection among vaccine recipients compared with that of the MenACWY control arm participants, derived by dividing the output curve from Fig. 1 by the overall risk of infection in the MenACWY control group. The green lines are the median relative risk obtained from 10,000 bootstrap samples. Green shaded areas are 95% bootstrapped CIs for the relative risk. The arrows point to the immune marker values at 0.20 and 0.50 relative risk, that is 80% and 50% VE for illustrative purpose. The full range of VE estimates from 50 to 90% are shown in Table 2.

**Fig. 3:**
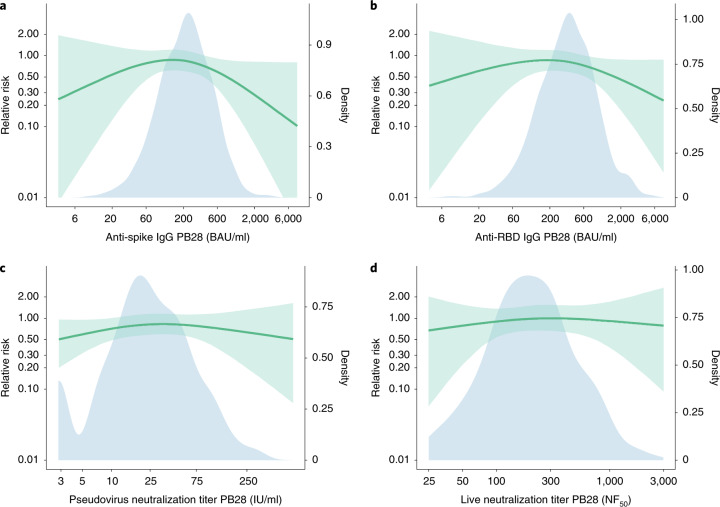
Relative risk of asymptomatic SARS-CoV-2 infection among vaccine recipients compared with the MenACWY control arm participants as a function of immune markers measured at PB28. **a**, Anti-spike IgG measured at PB28 (91 cases, 1,155 noncases included in the analysis). **b**, Anti-RBD IgG measured at PB28 (91 cases, 1,155 noncases included in the analysis). **c**, Pseudovirus neutralization antibody titers at PB28 (86 cases, 828 noncases included in the analysis). **d**, Live-virus neutralization antibody titers at PB28 (62 cases, 412 noncases included in the analysis). Blue shaded areas represent the immune marker density distribution. Green lines show the relative risk of infection among vaccine recipients compared with the MenACWY control arm participants. The green lines are the median relative risk obtained from 10,000 bootstrap samples. Green shaded areas are bootstrapped 95% CIs.

**Extended Data Fig. 4:**
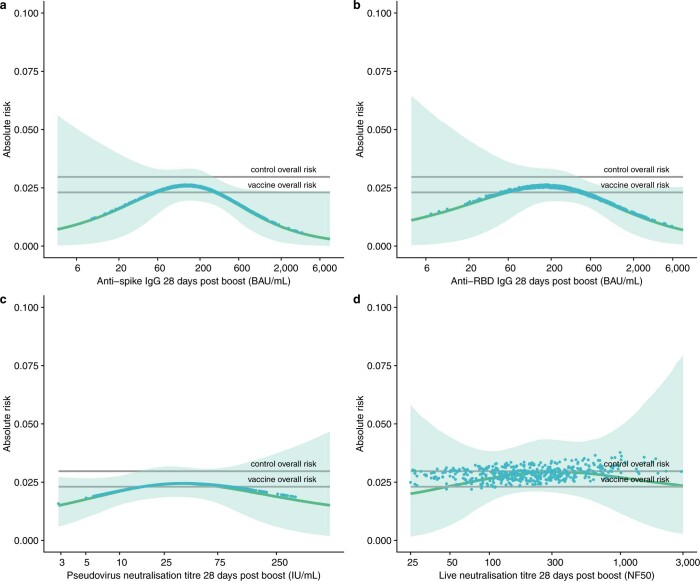
Predicted absolute risk of asymptomatic SARS-CoV-2 infection as a function of immune markers measured 28 days post second dose. Predicted absolute risk of asymptomatic infection as a function of: **a**: Anti-spike IgG measured at 28 days post boost (91 cases, 1155 non-cases included in the analysis). **b**: Anti-RBD IgG measured at 28 days post boost (91 cases, 1155 non-cases included in the analysis). **c**: Pseudovirus neutralisation antibody titres 28 days post boost (86 cases, 828 non-cases included in the analysis). **d**: Live-virus neutralisation antibody titres 28 days post boost (62 cases, 412 non-cases included in the analysis). Grey horizontal lines show the overall risk of primary symptomatic COVID-19 in the control group (MenACWY) and vaccine groups (ChAdOx1 nCoV-19). Blue dots show the absolute risk predicted from the model across the range of antibody values included in the analysis, adjusting for baseline exposure risk to SARS-CoV-2 infection. Green shaded areas show the confidence interval around the predicted mean probability (green line).

**Extended Data Fig. 5:**
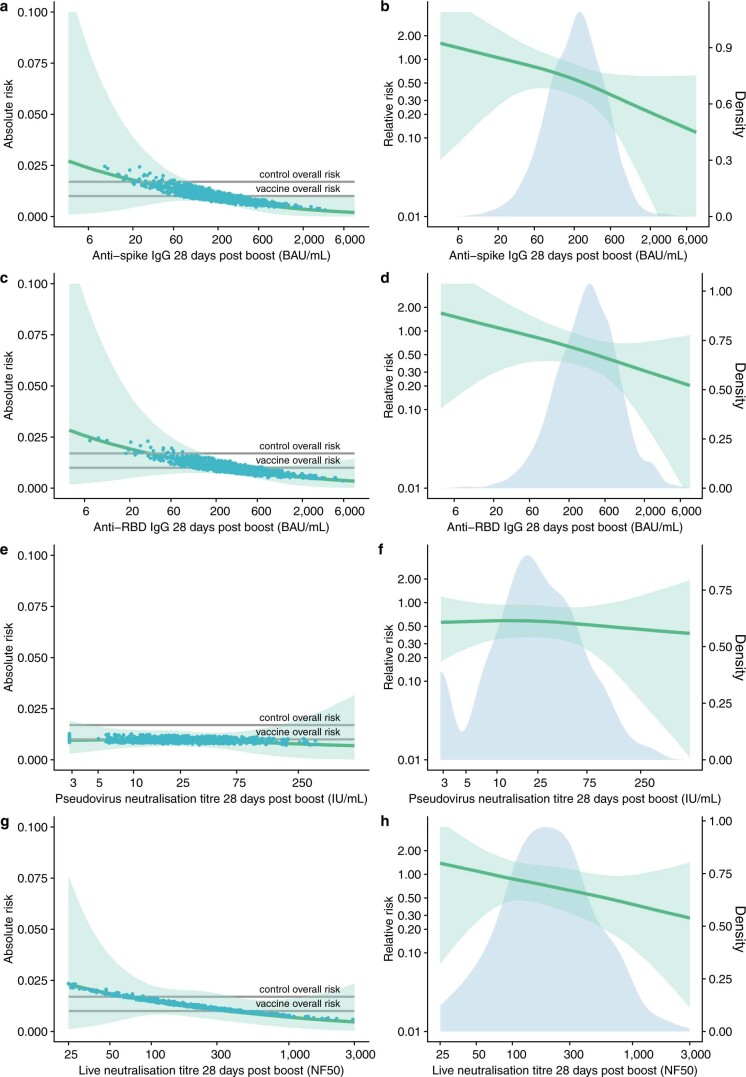
Sensitivity analysis showing absolute and relative risk of asymptomatic SARS-CoV-2 infection as a function of immune markers measured at 28 days post second dose excluding cases with low viral load (Ct ≥ 30). **a**, **c**, **e**, **g**: Grey horizontal lines show the overall risk of primary symptomatic COVID-19 in the control group (MenACWY) and vaccine groups (ChAdOx1 nCoV-19). Blue dots show the absolute risk predicted from the model across the range of antibody values included in the analysis, adjusting for baseline exposure risk to SARS-CoV-2 infection. Green shaded areas show the confidence interval around the predicted mean probability (green line). **b**, **d**, **f**, **h**: Blue shaded areas represent the immune marker density distribution. Green lines show the relative risk of infection among vaccine recipients compared to the MenACWY control arm participants. The green lines are the median relative risk obtained from 10,000 bootstrap samples. Green shaded areas are 95% bootstrapped confidence intervals for the relative risk.

**Extended Data Fig. 6:**
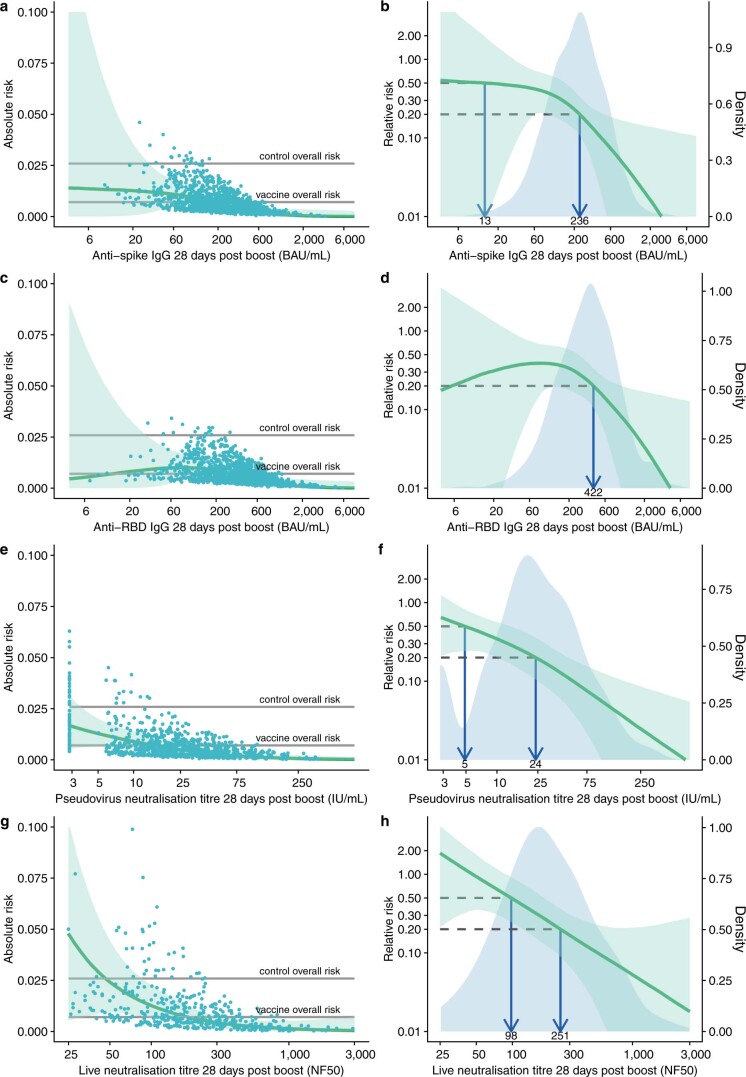
Sensitivity analysis showing absolute and relative risk of primary symptomatic SARS-CoV-2 infection in participants with symptoms of shortness of breath as a function of immune markers measured at day 28 post-second dose. Results are shown for: **a**, **b**: Anti-spike IgG measured at 28 days post boost (28 cases, 1155 non-cases included in the analysis). **c**, **d**: Anti-RBD IgG measured at 28 days post boost (28 cases, 1155 non-cases included in the analysis). **e**, **f**: Pseudovirus neutralisation antibody titres 28 days post boost (27 cases, 828 non-cases included in the analysis). **g**, **h**: Live virus neutralisation antibody titres 28 days post boost (22 cases, 412 non-cases included in the analysis). **a**, **c**, **e**, **g**: Grey horizontal lines show the overall risk of primary symptomatic COVID-19 in the control group (MenACWY) and vaccine groups (ChAdOx1 nCoV-19). Blue dots show the absolute risk predicted from the model across the range of antibody values included in the analysis, adjusting for baseline exposure risk to SARS-CoV-2 infection. Green shaded areas show the confidence interval around the predicted mean probability (green line) **b**, **d**, **f**, **h**: Blue shaded areas represent the immune marker density distribution. Green lines show the relative risk of infection among vaccine recipients compared to the MenACWY control arm participants. The green lines are the median relative risk obtained from 10,000 bootstrap samples. Green shaded areas are 95% bootstrapped confidence intervals for the relative risk.

**Extended Data Fig. 7:**
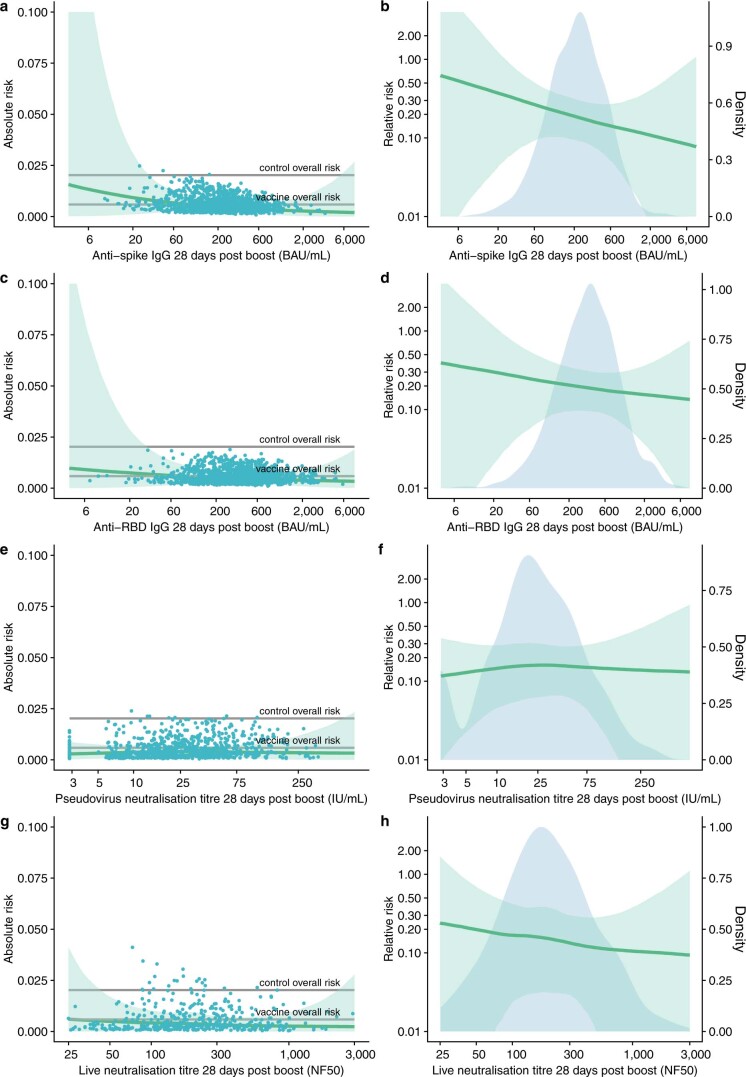
Sensitivity analysis showing absolute and relative risk of primary symptomatic SARS-CoV-2 infection in participants with no symptoms of shortness of breath as a function of immune markers measured at day 28 post-second dose. Results are shown for: **a**, **b**: Anti-spike IgG measured at 28 days post boost (24 cases, 1155 non-cases included in the analysis). **c**, **d**: Anti-RBD IgG measured at 28 days post boost (24 cases, 1155 non-cases included in the analysis). **e**, **f**: Pseudovirus neutralisation antibody titres 28 days post boost (20 cases, 828 non-cases included in the analysis). **g**, **h**: Live virus neutralisation antibody titres 28 days post boost (14 cases, 412 non-cases included in the analysis). **a**, **c**, **e**, **g**: Grey horizontal lines show the overall risk of primary symptomatic COVID-19 in the control group (MenACWY) and vaccine groups (ChAdOx1 nCoV-19). Blue dots show the absolute risk predicted from the model across the range of antibody values included in the analysis, adjusting for baseline exposure risk to SARS-CoV-2 infection. Green shaded areas show the confidence interval around the predicted mean probability (green line). **b**, **d**, **f**, **h**: Blue shaded areas represent the immune marker density distribution. Green lines show the relative risk of infection among vaccine recipients compared to the MenACWY control arm participants. The green lines are the median relative risk obtained from 10,000 bootstrap samples. Green shaded areas are 95% bootstrapped confidence intervals for the relative risk.

**Extended Data Fig. 8:**
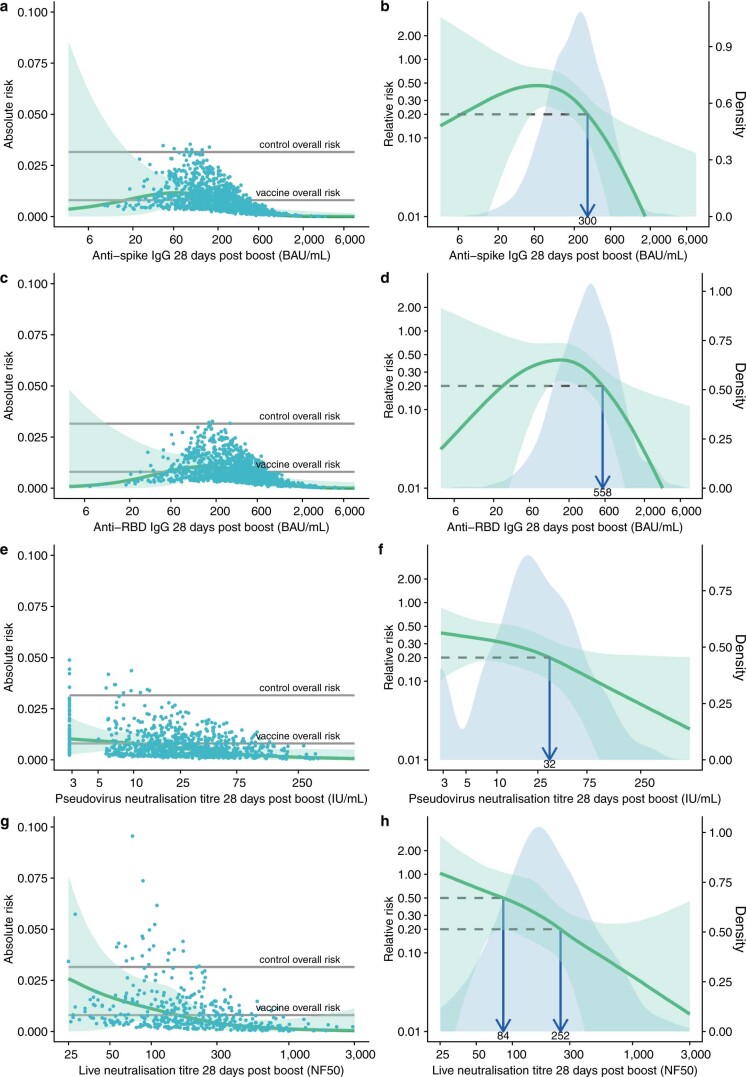
Sensitivity analysis showing absolute and relative risk primary symptomatic SARS-CoV-2 infection with 3 or more COVID-19 symptoms as a function of immune markers measured at day 28 post-second dose. Results are shown for: **a**, **b**: Anti-spike IgG measured at 28 days post boost (32 cases, 1155 non-cases included in the analysis). **c**, **d**: Anti-RBD IgG measured at 28 days post boost (32 cases, 1155 non-cases included in the analysis). **e**, **f**: Pseudovirus neutralisation antibody titres 28 days post boost (28 cases, 828 non-cases included in the analysis). **g**, **h**: Live virus neutralisation antibody titres 28 days post boost (21 cases, 412 non-cases included in the analysis). **a**, **c**, **e**, **g**: Grey horizontal lines show the overall risk of primary symptomatic COVID-19 in the control group (MenACWY) and vaccine groups (ChAdOx1 nCoV-19). Blue dots show the absolute risk predicted from the model across the range of antibody values included in the analysis, adjusting for baseline exposure risk to SARS-CoV-2 infection. Green shaded areas show the confidence interval around the predicted mean probability (green line). **b**, **d**, **f**, **h**: Blue shaded areas represent the immune marker density distribution. Green lines show the relative risk of infection among vaccine recipients compared to the MenACWY control arm participants. The green lines are the median relative risk obtained from 10,000 bootstrap samples. Green shaded areas are 95% bootstrapped confidence intervals for the relative risk.

The risk of symptomatic COVID-19 decreased with increasing levels of anti-spike IgG (*P* = 0.003), anti-RBD IgG (*P* = 0.018), pseudovirus neutralization titer (*P* = 0.005), and live-virus neutralization titer (*P* < 0.001) (Figs. 1 and 2 and Table 2). In contrast, there were no significant associations between any of the assays and protection against asymptomatic infection including for sensitivity analysis restricting to high viral load (all *P* > 0.05, Fig. 3, Extended Data Figs. 4, and 5, and Supplementary Table 4). When primary symptomatic COVID-19 cases were classified according to the presence of shortness of breath, we observed a similar trend, with increasing immune marker levels associated with lower risk of infection (all *P* < 0.05, Supplementary Table 4 and Extended Data Fig. 6), but not for those with no shortness of breath (all *P* > 0.05, Supplementary Table 4 and Extended Data Fig. 7). Higher pseudovirus and live-virus neutralization titers were associated with lower risk of infection for those who had three or more COVID-19 symptoms (Supplementary Table 4 and Extended Data Fig. 8). The number of cases and noncases included for correlates analysis by each immune marker and outcome has been summarized in Table 2 and Supplementary Table 4.

**Table 2:** Outputs from generalized additive models, with immune marker values associated with 50%, 60%, 70%, 80%, and 90% VE against symptomatic infection

Assay units	*P* value _immune marker_	*P* value _baseline risk score_	No. cases	No. noncase	50% VE (95% CI)	60% VE (95% CI)	70% VE (95% CI)	80% VE (95% CI)	90% VE (95% CI)
**Anti-spike IgG**									
AU/ml	0.003	<0.001	52	1155	4446 (NC, 12822)	8413 (NC, 22232)	17538 (NC, 37929)	40923 (16748, 125017)	139306 (57276, NC)
BAU/ml					29 (NC, 83)	54 (NC, 143)	113 (NC, 245)	264 (108, 806)	899 (369, NC)
**Anti-RBD IgG**									
AU/ml	0.018	<0.001	52	1155	2193 (NC, 13614)	6266 (NC, 29105)	20700 (NC, 56620)	63383 (16903, NC)	295781 (90567, NC)
BAU/ml					17 (NC, 109)	50 (NC, 232)	165 (NC, 452)	506 (135, NC)	2360 (723, NC)
**Normalized live-virus neutralization assay**									
NF_50_	<0.001	<0.001	36	412	68 (NC, 129)	91 (NC, 175)	135 (48, 267)	247 (101, NC)	938 (294, NC)
**Pseudovirus neutralization assay**									
ID_50_	0.005	<0.001	47	828	NC	22 (NC, 76)	57 (NC, 183)	185 (NC, NC)	982 (303, NC)
IU/ml					NC	3 (NC, 11)	8 (NC, 26)	26 (NC, NC)	140 (43, NC)

ID_50_^:^ neutralization dilution for 50% virus inhibition; NC: not computed; AU/ml: arbitrary units per mL; BAU/ml: binding antibody units per ml (WHO international standard 20/136), IU/ml: international units per ml (WHO international standard 20/136).

**Fig. 4:**
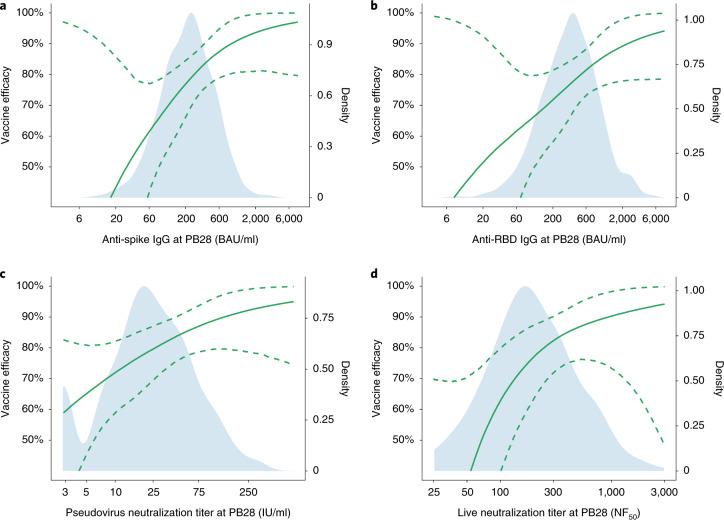
VE against primary symptomatic COVID-19 as a function of immune markers measured at PB28. **a**, Anti-spike IgG measured at PB28 (52 cases, 1,155 noncases included in the analysis). **b**, Anti-RBD IgG measured at PB28 (52 cases, 1,155 noncases included in the analysis). **c**, Pseudovirus neutralization antibody titers at PB28 (47 cases, 828 noncases included in the analysis). **d**, Live-virus neutralization antibody titers at PB28 (36 cases, 412 noncases included in the analysis). Blue shaded areas represent the immune marker density distribution. Green lines show the VE, and green dotted lines are 95% bootstrapped confidence intervals for VE. VE is computed as 1 minus the relative risks shown in Fig. 2. These results are also shown in Table 2 at 10% increments on the *y* axis.

Where CIs were outside the range of values of the assay the limits are reported as NC. VE estimates and CIs are those shown in Fig. 4, at every 10% increment in the *y* axis. The two-sided *P* value for each immune marker (column 2) is from the generalized additive models in Fig. 1, showing the strength of the relationship between the antibody value and infection. The *P* values were not adjusted for multiple comparisons.

The antibody level associated with 80% VE against primary symptomatic COVID-19 was 40,923 (95% CI: 16,748, 125,017) arbitrary units (AU)/ml for anti-spike IgG, equivalent to 264 BAU/ml (95% CI: 108, 806) using the WHO international standard (NIBSC code 20/136). For anti-RBD IgG, 80% efficacy was achieved with median antibody level of 506 (95% CI: 135, not computed (NC)) BAU/ml (Figs. 2 and 4 and Table 2).

For pseudovirus and live-virus neutralizing antibody titers, values of 26 (95% CI: NC, NC) IU/ml and 247 (95% CI: 101, NC) normalized neutralization titers (NF_50_), respectively, were associated with 80% VE against symptomatic infection (Table 2). No values from these assays were associated with protection against asymptomatic infection (Supplementary Table 4)

For all assays, when the analysis was restricted to symptomatic cases with shortness of breath, 80% VE was achieved at lower levels of immune markers than for symptomatic cases in general. Higher baseline exposure risk of SARS-CoV-2 infections predict higher probability of all outcomes (all *P* < 0.05, Table 2 and Supplementary Table 4), except for asymptomatic infections (*P* > 0.05) in generalized additive models.

## Discussion

Here, we report an analysis of potential correlates of protection using data from 171 cases of SARS-CoV-2 infection and 1,404 noncases, showing that higher anti-spike IgG, anti-RBD IgG, and neutralizing antibody titers are all associated with lower risk of symptomatic disease. We used immune responses in a phase 2/3 clinical trial to derive a model to predict absolute risk of infection, with appropriate adjustment for bias, assigning estimates for each level of antibody in the dataset. The relative risk of infection was then derived by reference to risk of infection in the control group. This is a robust approach to derive population estimates and was adapted from recently described methods^25,26^.

We previously published overall aggregate-level summaries of antibody levels in participants with different prime-boost intervals. Vaccination prime-boost intervals were associated with varying levels of VE, and there are some intriguing similarities between the aggregate-level data with the estimate provided from analysis of individual participant level data in this report. The estimated anti-spike IgG level of 40,923 AU/ml and the pseudovirus neutralizing antibody titer of 185 associated with 80% VE in our models were similar to the geometric mean titers of 48,961 AU/ml and 237.0, respectively, previously reported in the subgroup of participants vaccinated with ChAdOx1 nCoV-19 with a dose interval of at least 12 weeks between their first and second dose—a regimen that provided 80.0% (95% CI 65.2 to 88.5) VE in the pooled analysis of clinical trial data from the United Kingdom, Brazil, and South Africa^3^. The aggregate-level results previously published included all eligible participants in the assessment of VE, but only those with available antibody data were included in the summaries of immunogenicity, meaning that direct comparisons of efficacy with immunogenicity were not in the same populations. Our current approach analyzes the relationship between infections and antibody levels at the individual level in a single set of participants, with appropriate adjustment for confounding, providing robust outputs. In addition, the current work provides outputs in WHO standard units, which are necessary for comparisons with data from other laboratories with different assays.

In a preprint by Gilbert et al., correlates of protection derived from the Moderna phase 3 efficacy trial are reported using similar methodology^27^. Although overall binding and pseudovirus neutralizing antibody titers after vaccination were higher in that study than those measured after the ChAdOx1 nCoV-19 vaccine, the correlates of protection findings appear similar to those we report here.

No serological measurements in our data were shown to correlate with protection against asymptomatic infection or against symptomatic illness with only mild upper respiratory symptoms. This is consistent with our interim analysis that VE against asymptomatic infection was 27.3% (95% CI: −17.2 to 54.9) and was not significant at the 5% level^2^. These results are consistent with the real-world observation that infection remains possible in fully vaccinated individuals, despite high effectiveness against severe disease.

The antibody correlates presented in this report relate to protection against mild disease, defined as a NAAT+ test with at least one symptom present. Weekly self-swabbing in the trial enabled detection of many mild cases. At these antibody titers, efficacy against more severe endpoints, used in other trials, would be higher than the estimates in this analysis. Notably, this has been confirmed in the analysis of real-world effectiveness, in which the milder cases are not detected, after two doses of the vaccines in older adults in England, where VE was 90% for Pfizer and 89% for ChAdOx1 nCoV-19 against symptomatic disease using the same case definition for both vaccines^11^, while lower efficacy estimates were measured in our previously reported efficacy analysis with a milder disease endpoint^2^.

The correlates of vaccine efficacy reported here could be used to extrapolate efficacy to immunogenicity data for novel vaccines where clinical efficacy results are unavailable. A trial of a new vaccine that works through similar immune mechanisms and which produces antibody responses that are above the correlate values reported here in at least 50% of participants (that is, it has a similar or higher median), might be expected to have similar efficacy against the clinical endpoints used in our UK trial, and higher efficacy against more severe endpoints. We provide correlates for vaccine efficacy estimates ranging from 50% to 90% to allow flexibility in the way these estimates are utilized by the regulators and policy-makers.

It has previously been shown that protection against lower respiratory tract infection (LRTI) may be easier to achieve than against upper respiratory tract infection (URTI), as challenge studies in rhesus macaques have shown stronger correlation between neutralizing titers and the level of subgenomic messenger RNA in bronchoalveolar lavage samples than in nasal swab samples^28^.

Similarly, ChAdOx1 nCoV-19-vaccinated hamsters, with low neutralizing titers against B.1.351, were fully protected against LRTI following challenge with B.1.351, despite no evidence of protection against shedding of virus from the upper airway^29^. Protection against upper respiratory tract or asymptomatic infections may be more closely associated with the presence of secretory IgA on the mucosal surface which was not measured in this study^30^.

These observations indicate that the reduced neutralizing capacity against B.1.351 and other variants of concern, might drive reduced protection against initial infection, and perhaps transmission, but protection against severe disease is maintained. Clinical trials of SARS-CoV-2 vaccines have consistently shown higher efficacy against more severe forms of disease, such as those causing hospitalization or death, than against mild infections^2–5,15,31^. We are unable to assess correlates of protection against severe disease or hospitalization as there were no vaccinated participants hospitalized in the COV002 study.

Although live-virus and pseudovirus neutralization assays were modestly correlated with each other, the live-virus assay was more closely associated with protection against symptomatic COVID-19 than was the pseudovirus assay. This may reflect the sensitivity and dynamic range of the assays.

Protection against symptomatic COVID-19 is not absolute with any vaccine, and the results presented here show that there is no single threshold value for any of the assays investigated that was indicative of sterilizing immunity in our data. Instead, the probability of infection decreases on average with higher immune responses, but substantial variation exists between individuals. This is similar to studies of respiratory syncytial virus, in which risk of infection decreased with higher antibody levels, although infections were still observed at high antibody levels, suggesting that a definitive individual threshold of protection does not exist^32^. We provide antibody estimates that correspond with 50% to 90% VE; however, the wide CIs around these estimates should be noted.

These estimates represent the antibody level observed 28 days after a second dose of vaccine that provide protection during the subsequent 4- to 6-month period among UK COV002 efficacy and immunogenicity cohorts. This is different from the antibody level that would protect an individual at the time of exposure to the virus. Further work is needed to determine the durability of antibody and long-term protection after vaccination.

High levels of protection were noted after vaccination with one dose of a lipid nanoparticle RNA vaccine, despite modest levels of neutralizing antibody, strongly supporting the concept that other mechanisms are at play as co-correlates of protection^5,33^. We have previously shown that a wide range of Fc-mediated antibody functions are induced by vaccination, and it is possible that these functions may be important in the absence of neutralizing antibody^34^. Furthermore, strong T cell responses induced by ChAdOx1 nCoV-19 may contribute to protection^14,16^ and have been associated with recovery from COVID-19 disease^35–37^. The relationship between antibody and T cell responses may differ depending on the type of vaccine used, and care should be taken when interpreting data from clinical testing of different vaccine technologies.

There are some limitations to our analysis. These analyses are based on cases of COVID-19 detected in a mainly white population in the United Kingdom, which were mostly due to B.1.177 and B.1.1.7 variants. In settings in which these are not the dominant variants causing disease, or where neutralization assays use different strains of the virus, the modeled relationships between immune markers and disease outcomes shown here may not apply. In addition, we have conducted a large number of analyses, and therefore some caution should be taken when drawing conclusions on the basis of single *P* values alone as these are presented unadjusted for multiple comparisons. Furthermore, these analyses have been conducted on samples taken after two doses of ChAdOx1 nCoV-19 and might not apply to protection afforded by a single dose of the same vaccine or other COVID-19 vaccines. Correlates may also vary according to age profile, but this was not explored in our study due to the small number of older adults recruited. The potential role of T cells and interaction between humoral and cellular immunity has not been evaluated in this study. It is not possible to determine in this study if our results represent mechanistic or nonmechanistic correlates of protection, as many immune responses are highly correlated.

Correlates of protection can be used to bridge to new populations and new vaccines using validated assays. These data can be used to extrapolate efficacy estimates for new vaccines that use similar immune mechanisms and where efficacy data is unavailable.

## Methods

### Study description

The data included in this analysis comes from participants enrolled in COV002 (registration NCT04400838), a phase 2/3 randomized single-blind vaccine efficacy trial conducted across 19 sites in the United Kingdom. A full description of the trial including immunogenicity, efficacy, and safety data, and the protocol has been previously published^2,3,14,15,38^.

This study was approved in the United Kingdom by the Medicines and Healthcare products Regulatory Agency (MHRA), reference 21584/0428/001 0001, and the South-Central Berkshire Research Ethics Committee, reference 20/SC/0179. All participants provided informed consent.

Briefly, participants in the study were randomized to receive ChAdOx1 nCoV-19 or a MenACWY control vaccine. The randomization ratio (ChAdOx1 nCoV-19:MenACWY) differed by study cohort, and was either 1:1, 5:1, or 3:1. (see CONSORT diagram, Extended Data Fig. 1). Open label groups are not included in this report.

### Study endpoints and outcomes

Participants were reminded weekly to contact their study site if they experienced any of the primary symptoms of COVID-19 (fever ≥ 37.8 °C; cough; shortness of breath; anosmia or ageusia) and were assessed in clinic, with a nose and throat swab taken for NAAT. In addition, participants were asked to complete a nose and throat swab at home each week.

The outcomes for this analysis were (1) primary symptomatic COVID-19, that is a NAAT+ swab with at least one qualifying symptom, and (2) asymptomatic infections identified from weekly self-administered swabs, defined as a NAAT+ swab with no symptom reported. Sensitivity analysis of asymptomatic infections removed potential false-positive cases by restricting to those with higher viral load (cycle threshold (CT) value < 30). NAAT+ participants who had symptoms other than the main five COVID-19 symptoms were categorized as nonprimary symptomatic and were not included in correlates analysis.

Primary symptomatic COVID-19 outcomes were further classified according to whether a symptomatic participant reported shortness of breath or not, and whether three or more COVID-19 symptoms among five were present, indicators of more severe disease.

All endpoints were evaluated by a blinded independent clinical review committee.

### Immune markers and time points

A proportion of serum samples from vaccine recipients at PB28 were tested on 3 different assays with 4 assay readouts. All NAAT+ cases were tested if sample volume allowed, and a proportion of noncases were tested. Samples were tested blinded to case status. The data from noncases were obtained first, and consisted mainly of the samples processed for the initial application for emergency use which needed 15% of samples included in the efficacy cohort to be processed on validated assays. Subsequent to this NAAT+ cases were sent for testing as they occurred, if not already including the 15%. We assume the mechanism of missingness for samples that were not tested to be missing at random^39^. To account for the missing data, factors associated with sample availability were controlled as weights in the analysis (see ‘Correlates of risk’ and ‘Inverse probability weighting’ below).

Anti-SARS-CoV-2 Spike and RBD IgG were measured by a multiplex immunoassay on the MSD platform at PPD Laboratories. The assay sequences were based on the ancestral sequences from Wuhan, China. Antigen information and sequence information are provided in Supplementary Table 1. Assay validation included precision and ruggedness, dilutional linearity, selectivity, and relative accuracy for each SARS-CoV-2 antigens. Post-validation studies for stability and for conversion to the WHO standard, as well as the establishment of a cut-point, were performed. The lower limit of quantifications (LLOQs) for anti-spike and anti-RBD are 33 and 204 AU/ml, respectively.

Antibody neutralization was measured with a lentivirus-based pseudovirus particle expressing the D614 SARS-CoV-2 spike protein. The pseudovirus neutralizing antibody assay was validated at Monogram Biosciences. Validation included accuracy, repeatability, intermediate precision, linearity, specificity/selectivity, sensitivity, and stability utilizing pooled sera from high-titer, intermediate-titer, and low-titer pooled convalescent SARS-CoV-2 sera, as well as historical negative samples collected in the year 2017 (prior to SARS-CoV-2 circulation). The LLOQ for pseudovirus neutralizing antibody is 40 (ID_50_).

Antibody neutralization was also measured by a live microneutralization assay using the Victoria/01/2020 strain of the virus (Public Health England). Qualification of the assay included assessment of specificity, parallelism, dilutional linearity, repeatability, intermediate precision, and assessment of the assay range. A formal validation has since been completed (after the testing of clinical study samples in this manuscript). Normalized values (NF_50_) were used for the main analyses, as the normalization process removes the plate-to-plate variability and normalized values are more highly correlated with binding antibody and pseudovirus neutralization assays. However, normalized values cannot be converted into WHO standard units. A sensitivity analysis is provided in Supplementary Table 4 using non-normalized values (ND_50_), which are also presented as IU/ml using the WHO standard, but are less highly correlated with other assays. The LLOQ of the assay is 58 (ND_50_) and 8.6 (NF_50_).

Due to the limitations of laboratory capacity, fewer samples were tested for virus neutralization than were tested using the quicker multiplex assay.

#### Imputation on censored immune marker data in main analysis

Immune marker values were log_10_-transformed prior to analysis. Values that were censored at the lower limit of quantification (LLOQ) were imputed with the value LLOQ/2. Approximately 10% of the pseudovirus neutralization titer was censored at the LLOQ, and sensitivity analyses were conducted by imputing these values using a Gibbs sampler.

### Conversion to WHO International Standard (20/136)

Each assay was analyzed in its original scale, and results were then converted to the WHO international standard units using the conversion factors supplied by each laboratory. WHO standard units are BAU/ml for anti-spike and anti-RBD IgG, and IU/ml for neutralization titers^40^. For PPD conversion, factors are supplied with CIs. These are not able to be applied to the converted data, as it is a one-to-one conversion. For the Monogram assay, multiple forms of the conversion factor were supplied, and all three were implemented.

Conversion factors were as follows:

PPD: Conversion from AU/ml to BAU/ml

- Anti-spike IgG 0.00645, 95% CI (0.00594, 0.00701)
- Anti-RBD IgG 0.00798, 95% CI (0.00735, 0.00866)

Monogram pseudovirus neutralization assay (D614) conversion from ND_50_ to IU/ml

- 0.1428 (mean)
- 0.1458 (geometric mean)
- 0.1534 (median)

PHE live-virus neutralization assay conversion from ID_50_ to IU/ml

- 0.2461 (1/4.064)

### Study design and analysis populations

We first defined the correlates population by restricting it to participants who met the eligibility criteria and received ChAdOx1 nCoV-19: participants were eligible for inclusion if they were baseline seronegative to the SARS-CoV-2 N protein at first vaccination, had their PB28 visit within a 14- to 42-day window after the second dose, and were followed up to at least 7 days after PB28, with no prior evidence of infection. Participants were excluded from analysis if infection occurred before PB28. Participants who received two doses were included in the analysis, either standard dose followed by standard dose (SD/SD), or low dose followed by low or standard dose (LD/SD or LD/LD). Nine participants who received mixed schedules (one dose of ChAdOx1 nCoV-19 and one dose of MenACWY control) in error were excluded from analysis (Extended Data Fig. 1). The same eligibility criteria were applied to define a control population of MenACWY recipients.

Among the ChAdOx1 nCoV-19 correlates population, those who had biomarker data available comprised the correlates cohort. Participants who tested NAAT+ more than 7 days after PB28 were defined as cases, while those who did not have a positive test were defined as noncases. The 7-day window was implemented to exclude cases in which exposure is likely to have occurred before a blood sample was taken.

### Statistical Analysis

#### Baseline exposure risk to SARS-CoV-2 infections

To control for potential confounding due to variation in exposure risk among participants with available immune marker data, a logistic regression risk model was developed among the control population of MenACWY recipients. Baseline factors associated with exposure risk were used to model the probability of being NAAT+ in this population. Baseline variables for the risk model included age in years, ethnicity (white and nonwhite), BMI (<30 kg/m^2^, ≥30 kg/m^2^), comorbidities (having any of: respiratory disease; cardiovascular disease; or diabetes), and healthcare worker status (nonhealthcare worker, healthcare worker exposed to no more than one patient with COVID-19 on an average day; healthcare worker exposed to one or more patients with COVID-19 on an average day). Output is shown in Supplementary Table 3. The linear predictor from the risk model developed using the MenACWY control population was used to predict the baseline risk of exposure in the ChAdOx1 nCoV-19 correlates cohort.

#### Correlates or risk

The correlates of risk (CoR) analysis was conducted within the correlates cohort. log-transformed immune marker values were analyzed using generalized additive models (GAM) for binary data, with a cubic spline smooth applied to immune marker values to allow a nonlinear effect. The logit-transformed predicted baseline exposure risk was included as a linear covariate in the GAM model. A *P* value < 0.05 from the approximate significance test from the smooth GAM was used to determine if an immune marker was associated with protection. There was no adjustment for multiple comparison. Separate models were fitted for each immune marker controlling for baseline exposure risk, and weighted by inverse probability weights as described below.

#### Inverse probability weighting

Immune marker data were not available for everyone in the correlates population, and cases are over-represented in the immune marker datasets as these were preferentially processed over noncases. Unadjusted estimates of absolute risk of infection will therefore be inflated and result in bias to correlates estimates. We used a logistic regression model to predict the probability that a participant will have immune marker data available to the analysis. The outcome variables were each immune marker, and predictors were age group (18–55 years, 56–69 years, 70 years or above), whether the participant is a case or noncase, the type of case (primary symptomatic, nonprimary symptomatic, asymptomatic), prime-boost interval, and dosage (LD/LD, LD/SD, SD/SD). The inverse probability from this model was used to weight the correlates of risk models for each immune marker to remove this source of bias (Supplementary Table 3).

#### Correlates of vaccine efficacy

For each outcome, to derive the relative risk (RR) and correlates of vaccine efficacy, an estimate of the absolute averaged predicted risk from the CoR model was computed. The averaged absolute risk was then compared to the overall risk among MenACWY Correlates Population, which was itself weighted by the randomization ratio for study groups not randomized 1:1.

VE was defined as 100% × (1 – RR). Mean estimate of VE at each level of antibody in the dataset, as well as 95% CIs were calculated from 10,000 bootstrap samples.

Further analysis details are provided with the original trial statistical analysis plan (SAP) and the separate SAP developed for immune correlates analyses. The immune correlates SAP leant heavily on the methods proposed in the publicly available SAP by the Coronavirus Prevention Network (CoVPN) Biostatistics Team^26^.

#### Bootstrap

We resampled from all participants enrolled in the study. For each bootstrap sample, we calculated the inverse probability weights to account for sampling bias. We then estimated the CoR by GAM, adjusting for the baseline risk exposure and weighting by inverse probability weights. We compared the predicted absolute risk from the GAM across the full range of antibody values, with the resampled MenACWY control population weighted overall risk. Ten thousand bootstrap samples were used for each immune marker and outcome. The overall estimates for CoR and correlates of vaccine efficacy were given by the median value in the bootstrap; 95% CIs were calculated using the bootstrap percentile method, that is, the 2.5% and 97.5% quantiles from the bootstrap.

Correlates and their CIs were not computed for assays in which the relationship between antibody and outcome was nonsignificant. Where CIs were outside the range of values of the assay, these are reported as NC.

### Sensitivity analyses

#### Viral load

To account for potential of misclassification in asymptomatic infections, a sensitivity analysis was conducted excluding cases with lower viral loads (defined as those for whom all returned PCR positive tests had a CT value ≥ 30), as these are potential false positives.

#### Imputation of censored antibody values

Approximately 10% of the pseudovirus neutralization antibody titers were below the LLOQ. We performed a sensitivity analysis to account for the potential bias caused by imputing LLOQ / 2. Studies have shown that imputing LLOQ / 2 can lead to bias and CIs with poor coverage when a substantial proportion of the data are censored^41–43^. When a bootstrap is required for missing data, Brand et al. found single imputation embedded inside a bootstrap showed better statistical properties than other methods^43^. We used an iterative Gibbs sampler proposed by Chen et al. to impute the censored log pseudovirus neutralization antibody values^42^.

Not all participants with results from the pseudovirus neutralization titer also have results from the anti-spike, anti-RBD, and live neutralizing antibody titers. For each bootstrap sample, we iteratively predicted the missing and censored values for each antibody titers in a Gibbs sampler, constraining the predictions for the censored values to be less than or equal to the LLOQ. The antibody titers were iteratively predicted by a sequence of Bayesian linear regressions. For each regression, the independent variables were the current prediction for all other titers, the baseline risk score and all variables used in the inverse probability weighting model.

Let ${{{{Z}}}}_{{{{j}}}}$, j = 1, 2, 3, 4 be the vector of the *j*th antibody titers values. Let ${\sigma}_{{{{j}}}}^2$ and ${{{\mathbf{\beta }}}}_j$ be the variance and vector of regression parameters for the *j*th linear regression update, respectively. We chose a noninformative prior^42,44^ for ${\sigma}_{{{{j}}}}^2$ and ${{{\mathbf{\beta }}}}_j$, namely

$$p\left( {{\sigma}_{{{{j}}}}^2,{{{\mathbf{\beta }}}}_j} \right) \propto \frac{1}{{{\sigma}_{{{{j}}}}^2}}$$

Then the Gibbs sampler proposed by Chen et al. is as follows:^42^

Initialize the missing and censored values ${{{{Z}}}}_{{{{j}}}}^{(0)}$ for each ${{{{Z}}}}_{{{{j}}}}$, *j* = 1, 2, 3, 4.

For *i* = 1, …, *N*

For *j* = 1, 2, 3, 4

Update $\sigma _j^2$ and ${{{\mathbf{\beta }}}}_j$ from the posterior distribution given the current predictors for all other antibody values ${{{{Z}}}}_{{{\mathrm{k}}}}^{\left( i \right)}$, *k* < *j*; ${{{{Z}}}}_{{{\mathrm{k}}}}^{\left( {i - 1} \right)}$, k > *j* and the fixed covariates.

Update ${{{{Z}}}}_{{{{j}}}}^{\left( i \right)}$ from the posterior predictive distribution given $\sigma _j^2$ and ${{{\mathbf{\beta }}}}_j$ and the current values of the predictor variables.

We imputed a single value for each of the censored log pseudovirus neutralization antibody values from the *n* = 100th iteration of the Gibbs sampler. Note participants with missing log pseudovirus neutralization antibody titer values were excluded from the sensitivity analysis. The sensitivity analysis was then run on the imputed dataset for the bootstrap sample.

We initialized the Gibbs sampler by predicting the missing and censored values from a sequence of linear regressions on the nonmissing data. This sequence was developed with the data structure in mind, aiming to initialize the chain as close to the posterior mode as possible.

We ran multiple chains on bootstrap samples and tested for convergence by inspecting trace plots of the censored log pseudovirus neutralization titers. From these plots, we determined the 100th iteration to be approximately converged.

### Data cut-off

The data cut-off date for inclusion in this analysis was 28 February 2021. Cases occurring after this date are not included in the analysis.

### Software

Data analysis was done using R version 3.6.1 (ref. ^45^). The GAM was coded using the mgcv package^46^. Three knots were used for each GAM, and the smoothing parameter was estimated by generalized crossvalidation.

### Reporting Summary

Further information on research design is available in the Nature Research Reporting Summary linked to this article.

## Online content

Any methods, additional references, Nature Research reporting summaries, source data, extended data, supplementary information, acknowledgements, peer review information; details of author contributions and competing interests; and statements of data and code availability are available at 10.1038/s41591-021-01540-1.

## Supplementary information

Supplementary Information Supplementary Tables 1–4, R syntax including main functions for analyses, Acknowledgements, Statistical Analysis Plan on Immune Correlates Analysis

Reporting Summary

## Data Availability

Anonymized participant data will be made available when the trials are complete, upon requests directed to the corresponding author. Proposals will be reviewed and approved by the sponsor, investigator, and collaborators on the basis of scientific merit. After approval of a proposal, data can be shared through a secure online platform after signing a data access agreement. All data will be made available for a minimum of 5 years from the end of the trial.

## Code availability

The R code for the main correlates estimates is available in Supplementary File (item 5, page 8).
