# Supplementary material for: Correlates of protection against symptomatic and asymptomatic SARS-CoV-2 infection
Source: Nat Med. 2021 Sep 29;27(11):2032–40. doi: 10.1038/s41591-021-01540-1 (PMC8604724; doi:10.1038/s41591-021-01540-1)
Supplement: Supplementary file 1 — Supplementary Tables 1–4, R syntax including main functions for analyses, Acknowledgements, Statistical Analysis Plan on Immune Correlates Analysis [file 41591_2021_1540_MOESM1_ESM.pdf]

nature portfolio

https://doi.org/10.1038/s41591-021-01540-1

Supplementary information

Correlates of protection against symptomatic and asymptomatic SARS-CoV-2 infection

In the format provided by the authors and unedited

Correlates of protection against symptomatic and asymptomatic SARS-CoV-2 infection. Supplementary Material.

Table of Contents

1. **Table S1.** Antigen information and sequence information for SARS-CoV-2 Spike Trimer, SARS-CoV-2 Nucleocapsid and SARS-CoV-2 Receptor Binding Domain……**Pg. 2**
2. **Table S2**. Anti-SARS-CoV-2 Spike and RBD IgG, pseudovirus neutralisation antibody titres and live virus neutralisation antibody titres (Median and IQR) by primary symptomatic, asymptomatic/unknown, non-primary cases, NAAT positive cases and negative controls…………………………………………………………………………………………**Pg. 3**
3. **Table S3**. Coefficients and p-values from baseline exposure logistic regression model fitted to MenACWY control participants, and from the inverse probability weighting model for predicting the availability of anti-spike IgG antibody data……………**Pg. 4**
4. **Table S4.** Outputs from modelling of correlates of vaccine efficacy, with immune marker values associated with vaccine efficacy estimates at 10% increments ……………………………………………………………………………………………………**Pg. 5**
5. R syntax including main functions for analyses ……………………………………………**Pg. 8**
6. Acknowledgements…………………………………………………………………………………**Pg. 10**

Table S1. Antigen information and sequence information for SARS-CoV-2 Spike Trimer, SARS-CoV-2 Nucleocapsid and SARS-CoV-2 Receptor Binding Domain

Antigen information:
Antigen Name: SARS-CoV-2 Spike Trimer
Organism: SARS-CoV-2 (Novel Coronavirus 2019-nCoV)
MSD Spot Name: CoV-2 Spike

Sequence Information:
Protein Sequence:
MFVFLVLLPLVSSQCVNLTTRTQLPPAYTNSFTRGVYYPDKVFRSSVLHSTQDLFLPFFSNVTWFHAIHVSGTNGTKRFDN
PVLPFNDGVYFASTEKSNIIRGWIFGTTLDSKTQSLLIVNNATNVVIKVCEFQFCNDPFLGVYYHKNNKSWMESEFRVYSS
ANNCTFEYVSQPFLMDLEGKQGNFKNLREFVFKNIDGYFKIYSKHTPINLVRDLPQGFSALEPLVDLPIGINITRFQTLLALH
RSYLTPGDSSSGWTAGAAAYYVGYLQPRTFLLKYNENGTITDAVDCALDPLSETKCTLKSFTVEKGIYQTSNFRVQPTESIV
RFPNITNLCPFGEVFNATRFASVYAWNRKRISNCVADYSVLYNSASFSTFKCYGVSPTKLNDLCFTNVYADSFVIRGDEVR
QIAPGQTGKIADYNYKLPDDFTGCVIAWNSNNLDSKVGGNYNYLYRLFRKSNLKPFERDISTEIYQAGSTPCNGVEGFNC
YFPLQSYGFQPTNGVGYQPYRVVVLSFELLHAPATVCGPKKSTNLVKNKCVNFNFNGLTGTGVLTESNKKFLPFQQFGRD
IADTTDAVRDPQTLEILDITPCSFGGVSVITPGTNTSNQVAVLYQDVNCTEVPVAIHADQLTPTWRVYSTGSNVFQTRAG
CLIGAEHVNNSYECDIPIGAGICASYQTQTNSPGSASSVASQSIIAYTMSLGAENSVAYSNNSIAIPTNFTISVTTEILPVSMT
KTSVDCTMYICGDSTECSNLLLQYGSFCTQLNRALTGIAVEQDKNTQEVFAQVKQIYKTPPIKDFGGFNFSQILPDPSKPSK
RSFIEDLLFNKVTLADAGFIKQYGDCLGDIAARDLICAQKFNGLTVLPPLLTDEMIAQYTSALLAGTITSGWTFGAGAALQI
PFAMQMAYRFNGIGVTQNVLYENQKLIANQFNSAIGKIQDSLSSTASALGKLQDVVNQNAQALNTLVKQLSSNFGAISS
VLNDILSRLDPPEAEVQIDRLITGRLQSLQTYVTQQLIRAAEIRASANLAATKMSECVLGQSKRVDFCGKGYHLMSFPQSA
PHGVVFLHVTYVPAQEKNFTTAPAICHDGKAHFPREGVFVSNGTHWFVTQRNFYEPQIITTDNTFVSGNCDVVIGIVNN
TVYDPLQPELDSFKEELDKYFKNHTSPDVDLGDISGINASVVNIQKEIDRLNEVAKNLNESLIDLQELGKYEQSAGYIPEAPR
DGQAYVRKDGEWVLLSTFLGENLYFQGHHHHHHGSAWSHPQFEKGGGSGGGSGGSAWSHPQFEK

Antigen information:
Antigen Name: SARS-CoV-2 Nucleocapsid
Organism: SARS-CoV-2 (Novel Coronavirus 2019-nCoV)
MSD Spot Name: CoV-2 N

Sequence Information:
Protein Sequence:
MKWVTFISLLFLFSSAYSRGSDNGPQNQRNAPRITFGGPSDSTGSNQNGERSGARSKQRRPQGLPNNTASWFTALTQH
GKEDLKFPRGQGVPINTNSSPDDQIGYYRRATRRIRGGDGKMKDLSPRWYFYYLGTGPEAGLPYGANKDGIIWVATEG
ALNTPKDHIGTRNPANNAAIVLQLPQGTTLPKGFYAEGSRGGSQASSRSSSRSRNSSRNSTPGSSRGTSPARMAGNGGD
AALALLLLDRLNQLESKMSGKGQQQQGQTVTKKSAAEASKKPRQKRTATKAYNVTQAFGRRGPEQTQGNFGDQELIR
QGTDYKHWPQIAQFAPSASAFFGMSRIGMEVTPSGTWLTYTGAIKLDDKDPNFKDQVILLNKHIDAYKTFPPTEPKKDK
KKKADETQALPQRQKKQQTVTLLPAADLDDFSKQLQQSMSSADSTQAENLYFQGSHHHHHHP

Antigen information:
Antigen Name: SARS-CoV-2 Receptor Binding Domain
Organism: SARS-CoV-2 (Novel Coronavirus 2019-nCoV)
MSD Spot Name: CoV-2 RB

Sequence Information:
Protein Sequence: R319-F541 of the SARS-2 CoV Spike Sequence
QRVQPTESIVRFPNITNLCPFGEVFNATRFASVYAWNRKRISNCVADYSVLYNSASFSTFKCYGVSPTKLNDLCFTNVYA
DSFVIRGDEVRQIAPGQTGKIADYNYKLPDDFTGCVIAWNSNNLDSKVGGNYNYLYRLFRKSNLKPFERDISTEIYQAGS
TPCNGVEGFNCYFPLQSYGFQPTNGVGYQPYRVVVLSFELLHAPATVCGPKKSTNLVKNKCVNFHHHHHH

Table S2. Anti-SARS-CoV-2 Spike and RBD IgG, pseudovirus neutralisation antibody titres and live virus neutralisation antibody titres (Median and IQR) by primary symptomatic, asymptomatic/unknown, non-primary cases, NAAT positive cases and negative controls

Immune marker	Outcome	No. participant	Median (IQR)
**Anti-spike IgG (AU/ml)**	**NAAT Negative**	1155	33945 (18450, 59260)
	**NAAT positive**	163	30501 (16088, 49529)
	Primary	52	26144 (16147, 39996)
	Asymptomatic	91	31115 (16112, 54118)
	Non-primary	20	33896 (15976, 45307)
**Anti-RBD IgG (AU/ml)**	**NAAT Negative**	1155	45693 (24009, 82432)
	**NAAT positive**	163	40884 (20871, 62934)
	Primary	52	37276 (21560, 58033)
	Asymptomatic	91	40884 (20944, 74226)
	Non-primary	20	43673 (20474, 54332)
**Pseudovirus neutralisation titre (ID_{50})**	**NAAT Negative**	828	158 (81, 328)
	**NAAT positive**	149	160 (85, 304)
	Primary	47	135 (75, 240)
	Asymptomatic	86	172 (91, 338)
	Non-primary	16	162 (109, 260)
**Live virus neutralisation titre (NF_{50})**	**NAAT Negative**	412	184 (101, 344)
	**NAAT positive**	110	206 (124, 331)
	Primary	36	166 (112, 231)
	Asymptomatic	62	261 (129, 359)
	Non-primary	12	176 (138, 277)

Primary symptomatic cases: NAAT+ with at least one COVID symptom (cough, fever, shortness of breath, anosmia, aguesia). Asymptomatic cases: NAAT+ on weekly self-swab with no symptoms recorded. Non-primary cases: NAAT+ with only non-primary COVID symptoms (e.g. nausea, diarrhoea). ND_{50}: neutralisation dilution for 50% virus inhibition; NF_{50}: Normalised ND50 values; IgG: Immunoglobulin G; RBD: receptor binding domain. AU/ml: arbitrary units per millilitre.

Table S3. Coefficients and p-values from baseline exposure logistic regression model fitted to MenACWY control participants, and from the inverse probability weighting model for predicting the availability of anti-spike IgG antibody data.

	Coefficient from logistic regression (log-odds ratio)	p-value
**Baseline Exposure Risk Model**		
**Age (per year)**	-0.0260	<0.001
**Non-white ethnicity (vs white)**	-0.0885	0.702
**BMI ≥ 30 (vs < 30 kg/m^2)**	0.1322	0.348
**Having any defined co-morbidities (vs none)**	-0.0935	0.514
**Healthcare worker status**		
Not a healthcare worker	ref	
Healthcare worker facing more than 1 COVID patient per day	0.3418	0.017
Healthcare worker facing up to 1 COVID patient per day	0.1379	0.331
**Inverse Probability Weighting Model for Anti-spike IgG**		
**Age group**		
18-55 years	ref	
56-69 years	-0.7515	<0.001
≥70 years	-1.0705	<0.001
**Dosage schedule**		
LD/LD	ref	
LD/SD	17.0832	0.927
SD/SD	17.7413	0.925
**Prime-boost interval**		
< 6 weeks	ref	
6-8 weeks	-0.1096	0.504
9-11 weeks	-0.3195	0.071
≥12 weeks	-0.8142	<0.001
**Outcome**		
NAAT negative	ref	
Primary symptomatic	4.7880	<0.001
Asymptomatic	4.4852	<0.001
Non-primary symptomatic	17.4349	0.973

The p values were obtained by two-sided Wald test.

Table S4. Outputs from modelling of correlates of vaccine efficacy, with immune marker values associated with vaccine efficacy estimates at 10% increments

Unit	Outcome	p immune marker	p baseline risk score	No. case	No. Non case	50% VE (95% CI)	60% VE (95% CI)	70% VE (95% CI)	80% VE (95% CI)	90% VE (95% CI)
**Anti-Spike IgG**										
	Asymptomatic	0.215	0.951	91	1155					
	Asymptomatic (Ct < 30)	0.059	0.375	39	1207					
**AU/ml[a]**	Shortness of breath	0.016	0.001	28	1155	2075 (11349, 23658)	11402 (11349, 30901)	21876 (11349, 40829)	36641 (14092, 76203)	70464 (38634, 1629184)
**BAU/ml[a]**	Shortness of breath	0.016	0.001	28	1155	13 (73, 153)	74 (73, 199)	141 (73, 263)	236 (91, 492)	454 (249, 10508)
	No shortness of breath	0.238	0.006	24	1155					
**AU/ml[a]**	3 or more symptoms	0.014	0.001	32	1155	NC	20796 (NC, 39081)	31693 (NC, 51876)	46448 (24545, 89119)	77619 (45917, 394430)
**BAU/ml[a]**	3 or more symptoms	0.014	0.001	32	1155	NC	134 (NC, 252)	204 (NC, 335)	300 (158, 575)	501 (296, 2544)
**Anti-RBD IgG**										
	Asymptomatic	0.283	0.905	91	1155					
	Asymptomatic (Ct < 30)	0.083	0.391	39	1207					
**AU/ml[a]**	Shortness of breath	0.035	0.002	28	1155	NC	NC	31115 (NC, 59425)	52841 (NC, 126465)	103269 (55204, NC)
**BAU/ml[a]**	Shortness of breath	0.035	0.002	28	1155	NC	NC	248 (NC, 474)	422 (NC, 1009)	824 (441, NC)

	No shortness of breath	0.494	0.009	23	1155					
**AU/ml[a]**	3 or more symptoms	0.032	0.002	32	1155	NC	28838 (NC, 58340)	46663 (NC, 81465)	69979 (34671, 162170)	119942 (67759, 1462076)
**BAU/ml[a]**						NC	230 (NC, 466)	372 (NC, 650)	558 (277, 1294)	957 (541, 11667)
**Live virus neutralisation assay**										
**ND50**	Symptomatic	<0.001	<0.001	36	414	167 (35, 282)	209 (83, 364)	290 (143, 537)	488 (229, 1211)	4188 (538, 1211)
**IU/ml**	Symptomatic					41 (9, 69)	52 (20, 90)	71 (35, 132)	120 (56, 298)	1030 (132, 298)
	Asymptomatic	0.591	0.345	62	412					
	Asymptomatic (Ct < 30)	0.068	0.802	29	445					
**NF50**	Shortness of breath	<0.001	<0.001	22	412	98 (52, 184)	124 (52, 228)	167 (78, 310)	251 (122, NC)	512 (233, NC)
	No shortness of breath	0.482	<0.001	14	412					
**NF50**	3 or more symptoms	<0.001	<0.001	21	412	84 (NC, 209)	118 (NC, 242)	168 (NC, 324)	252 (NC, 643)	495 (217, 643)
**Pseudovirus neutralisation assay**										
**ID50 [b]**	Symptomatic	0.005	<0.001	47	828	13 (NC, 42) [b]	25 (NC, 69) [b]	61 (NC, 167) [b]	187 (NC, 1455) [b]	1021 (299, NC) [b]
**IU/ml[c]**						NC	3 (NC, 11)	8 (NC, 27)	27 (NC, NC)	143 (44, NC)
**IU/ml[d]**						NC	3 (NC, 12)	9 (NC, 28)	28 (NC, NC)	151 (47, NC)
	Asymptomatic	0.404	0.998	86	828					

	Asymptomatic (Ct < 30)	0.785	0.486	37	877					
**ID50**						34 (NC, 96)	54 (NC, 142)	90 (NC, 208)	167 (71, 348)	386 (177, 1230)
**ID50 [b]**	Shortness of breath	<0.001	<0.001	27	828	37 (NC, 84) [b]	55 (NC, 124) [b]	90 (NC, 186) [b]	162 (69, 327) [b]	385 (171, 1337) [b]
**IU/ml**						5 (NC, 14)	8 (NC, 20)	13 (NC, 30)	24 (10, 50)	55 (25, 176)
	No shortness of breath	0.868	<0.001	20	828					
**ID50**						NC	23 (NC, 176)	89 (NC, 284)	228 (NC, 7295)	644 (232, NC)
**ID50 [b]**	3 or more symptoms	0.015	<0.001	28	828	NC [b]	NC [b]	99 (NC, 280) [b]	236 (NC, 2198) [b]	625 (232, NC) [b]
**IU/ml**						NC	3 (NC, 25)	13 (NC, 41)	32 (NC, 1042)	92 (33, NC)

ID50 and ND50: neutralisation dilution for 50% virus inhibition; NF50: Normalised ND50 values; NC: not computed; IgG: Immunoglobulin G; RBD: receptor binding domain. AU/ml: arbitrary units per millilitre; BAU/ml: Binding antibody units per millilitre (WHO international standard 20/136), IU/ml: international units per millilitre (WHO international standard 20/136).

Results were not computed for assays in which the relationship between antibody and outcome was non-significant. Where CIs were outside the range of values of the assay these are reported as 'not computed' (NC).The two-sided p value for each immune marker (column 3) is from the generalised additive models, showing the strength of the relationship between the antibody value and infection. The p-values were not adjusted for multiple comparisons.

[a] BAU/ml and IU/ml values are presented using the WHO international standard (NIBSC code 20/136).

[b] Sensitivity analysis using a Gibbs sampler within each bootstrap sample to impute censored pseudovirus neutralisation titres below LLOQ.

[c] Alternative WHO conversion using geometric mean conversion factor rather than the mean conversion factor shown in Table 2

[d] Alternative WHO conversion using median conversion factor rather than the mean conversion factor shown in Table 2

R syntax including main functions for analyses

```
#
# R syntax including main functions for analyses on September 6th 2021
#
# Shuo Feng, Daniel Phillips, Thomas White, Homesh Sayal, et al.
# Correlates of protection against symptomatic and asymptomatic SARS-CoV-2 infection
#
# Writen by Shuo Feng
#
# This function is used for estimating the point estimates of correlates of risk for each participant in dataset
# Median and 95% CI could be further obtained by bootstrap resampling, which is not covered by this function
#
# Arguments in the function f.cor.PB28.ipw.bs
# data - Main dataset for correlates analysis
# model.predictor - binding and neutralising antibody variables (log scale)
# model.outcome - symptomatic/asymptomatic SARS-CoV-s infection variables
#

f.cor.PB28.ipw.bs <- function(data, model.predictor, model.outcome){

  rand.ratio1 <- 3 # randomisation ratio 3:1 for study group 1 and 7 in COV002
  rand.ratio2 <- 5 # randomisation ratio 5:1 for study group 2, 8  and 5d in COV002

  # model variable - predictor and outcome
  data$pred.var <- data[, model.predictor]
  data$outcome.var <- data[, model.outcome]

  # get weighted incidences among control participants receiving MenACWY vaccine
  tab <- as.data.frame.matrix(table(data$cor2dose_outcome[which(data$As_vaccinated_arm_2 == 'Control',)],
                              data$study_group_2[which(data$As_vaccinated_arm_2 == 'Control',)]))
  tab <- as.data.frame(t(tab))
  tab$study_group_2 <- rownames(tab)
  tab[which(tab$study_group_2 %in% c(1,7)), 1:4] <- tab[which(tab$study_group_2 %in% c(1,7)),
1:4]*rand.ratio1
  tab[which(tab$study_group_2 %in% c(2,8,'5d')), 1:4] <- tab[which(tab$study_group_2 %in% c(2,8,'5d')),
1:4]*rand.ratio2
  tab <- data.frame(rbind(tab, c(colSums(tab[,1:4]), 'total')))
  colnames(tab) <- c('negative', 'symptomatic', 'asymptomatic', 'non-primary', 'study_group_2')

  # restrict participants to Correlates Population (both ChAdOx and MenACWY)
  data <- data[which(data$cor2dose_cohort == 'Yes'), ]

  # incidence table by ChAdOx and MenACWY control group, before and after weighting
  inc <- data.frame(n.total = c(sum(data$As_vaccinated_arm_2 == 'ChAdOx1' & !is.na(data[, model.outcome])),
                         sum(data$As_vaccinated_arm_2 == 'Control' & !is.na(data[, model.outcome]))))
  inc$cases <- c(sum(data$As_vaccinated_arm_2 == 'ChAdOx1' & data[, model.outcome] == 1, na.rm = T),
            sum(data$As_vaccinated_arm_2 == 'Control' & data[, model.outcome] == 1, na.rm = T))
  inc$non_cases <- c(sum(data$As_vaccinated_arm_2 == 'ChAdOx1' & data[, model.outcome] == 0, na.rm = T),
              sum(data$As_vaccinated_arm_2 == 'Control' & data[, model.outcome] == 0, na.rm = T))
  inc$abs_risk <- inc$cases/(inc$cases + inc$non_cases)
  rownames(inc) <- c('ChAdOx1', 'Control')
  inc$n.total_w <- inc$n.total # weighted total
  inc$n.total_w[2] <- as.numeric(tab$negative[nrow(tab)]) + as.numeric(tab[nrow(tab), model.outcome]) #
weighted total account for weights in MenACWY
  inc$cases_w <- inc$cases # weighted number of cases
```

```
  inc$cases_w[2] <- as.numeric(tab[nrow(tab), model.outcome]) # weighted number of cases account for
weights in MenACWY
  inc$non_cases_w <- inc$n.total_w - inc$cases_w # weighted number of non-cases
  inc$abs_risk_w <- inc$cases_w/inc$n.total_w

  # restrict to ChAdOx Correlates Population
  data <- data[which(data$As_vaccinated_arm_2 == 'ChAdOx1'), ]

  # Inverse probability weighting model on antibody sampling
  data$antibody <- 1*(!is.na(data$pred.var))
  m.s <- glm(antibody ~  age.group + endpoint.dose + interval.prime.cutoff + cor2dose_outcome,
         family = 'binomial', data = data)
  data$p_s <- predict(m.s, newdata = data, type = 'response')
  data <- data[which(data$outcome.var == 1 | data$positive == 0), ]

  # fit GAM using inverse probability weights
  data.pred <- data[which(!is.na(data$pred.var) & !is.na(data$outcome.var) & !is.na(data$risk_score_2)), ]
  data.pred <- data.pred[order(data.pred$pred.var), ]

  m2 <- gam(outcome.var ~ s(pred.var, bs="cr", k = 3) + risk_score_2, data = data.pred, family = "binomial",
weights = 1/p_s)

  output <- list(inc = inc,
              model2 = summary(m2)) # output incidence table and GAM model

  # predict absolute risk
  pm2 <- data.frame(antibody = data.pred$pred.var) # antibody in original dataset
  pm2$pred <- predict(m2, newdata = data.pred, type = 'response') # predicted absolute risk for each individual
participants
  output$pm2 <- pm2 # output prediction results

  return(output)
}
```

Acknowledgements

Clinical Trials Research Governance Office, University of Oxford
Ronja Bahadori
Elaine Chick
Heather House
Claire Riddle
**Data and Safety Monitoring Board (DSMB)**
George Bouliotis
Steve Black
Elizabeth Bukusi
Cornelia Dekker
Robert Heyderman
Gregory Hussey
Paul Kaye
Bernhards Ogutu
Walter Orenstein
Sonia Ramos
Manish Sadarangani
**Department of Paediatrics, University of Oxford**
Georg A. Holländer
**Endpoint Evaluation Committee**
Jeremy Carr
Steve Chambers
Kim Davis
Simon Drysdale
Malick Gibani
Elizabeth Hammershaimb
Michael Harrington
Celina Jin
Seilesh Kadambari
Rama Kandasamy
Toby Maher
Jamilah Meghji
Claire Munro
David Pace
Rekha Rapaka
Robindra Basu Roy
Daniel Silman
Gemma Sinclair
Jing Wang
**Jenner Institute, University of Oxford**
Iona Tarbet
**Nuffield Department of Medicine, University of Oxford**
Richard Cornall
Richard Liwicki

Denis Murphy
Elizabeth Salter
Katherine Skinner
Philip Taylor
Oto Velicka
**Oxford Research Services (Contracts)**
Carly Banner
Sally Pelling-Deeves
Gary Priest
**Oxford University Hospitals Trust**
Bruno Holthof
**Public Affairs Directorate and Divisional Communication Team**
Alison Brindle
Alexander Buxton
James Colman
Chris McIntyre
Steve Pritchard

**Statistical Analysis Plan- Correlates Analysis**	
Study Code	D8111
Edition Number	1.0
Date	28 May 2021

Immune Correlates Analysis

TABLE OF CONTENTS

TITLE PAGE.....1
TABLE OF CONTENTS.....2
LIST OF ABBREVIATIONS.....3
AMENDMENT HISTORY.....4
1. INTRODUCTION.....5
1.1 Study Cohort for Correlates Analyses.....5
1.1.1 Single Dose Analyses.....5
1.1.2 Two Dose Analyses.....5
1.2 Study Endpoints.....6
1.3 Sample Size.....6
1.4 Immune Markers.....6
2. ANALYSIS.....6
2.1 Data summaries.....6
2.2 Risk model (Proxy for SARS-CoV-2 Exposure in participants who received control vaccine).....7
2.3 Risk model for availability of antibody data (AZD1222 arm).....7
2.4 Correlates of Risk.....8
2.5 Correlates of Vaccine Efficacy (CoVE).....8
2.6 Imputation of Censored Data.....8
3. REFERENCES.....9

LIST OF FIGURES

Figure 1 Example plot of predicted probabilities against antibody values.....8

LIST OF ABBREVIATIONS

**Abbreviation or special term**	**Explanation**
BMI	body mass index
ChAdOx1 nCoV-19	name of AZD1222 by the University of Oxford
COVID-19	coronavirus disease 2019
GAM	generalised additive model
GLM	generalised linear model
IgG	immunoglobulin G
LD	low dose
LD/LD	low dose low dose
LD/SD	low dose standard dose
LLOQ	lower limit of quantification
MAA	Marketing Authorisation Application
MenACWY	meningococcal Group A, C, W-135 and Y conjugate vaccine
PHE	Public Health England
RBD	receptor binding domain
RT-PCR	reverse transcriptase polymerase chain reaction
SAP	statistical analysis plan
SARS-CoV-2	severe acute respiratory syndrome-coronavirus 2
SD	standard dose
SD/SD	standard dose standard dose
WHO	World Health Organization

AMENDMENT HISTORY

Version 1: Summary of Changes

Category[a]: Change refers to	Date	Description of change	Rationale
Not applicable	28 May 2021	Not applicable – first version	Not applicable

[a] Pre-specified categories are: Primary or secondary endpoints; Statistical analysis method for the primary or secondary endpoints; Derivation of primary or secondary endpoints; Multiple Testing Procedure; Data presentations; Other

1. INTRODUCTION

The Statistical Analysis Plan (SAP) describes the statistical analysis of immune markers measured at key time points post-vaccination as correlates of protection against COVID-19.

This document references concepts published in "CoVPN COVID-9 Vaccine Efficacy Trial Immune Correlates SAP" by the CoVPN Biostatistics Team. Version 0.1, November 9, 2020.

1.1 Study Cohort for Correlates Analyses

Blood samples were taken 28±14 days (14 to 42 days) after the first dose and 28±14 days (14 to 42 days) after a second dose in vaccine recipients. An additional 7-day window (35 day timepoint; case date > blood sample collection date + 7) is implemented to exclude cases in which exposure is likely to have occurred before a blood sample was taken.

1.1.1 Single Dose Analyses

The Single Dose for Correlates population is defined as AZD1222 recipients who received a first standard dose (SD) or low dose (LD) vaccine, having follow-up time accrued at least 7 days after their day 28 blood sample visit after a first vaccine.

Cases will be those which meet study endpoint definitions as detailed below and occur more than 7 days after the day 28 visit (+/- 14 days) after a first SD or LD vaccine and before a booster dose in the Single Dose for Correlates population. Participants with evidence of infection prior to day 35 will be excluded.

The control cohort consists of participants in the Single Dose for Correlates population who have no evidence of infection with COVID-19 up to the data cut-off date for analysis, and for whom immune marker data is available for analysis.

1.1.2 Two Dose Analyses

Two Dose for Correlates population is defined as AZD1222 recipients who received SD/SD, LD/SD, SD/LD, LD/LD vaccines, having follow-up time accrued until at least 7 days after their boost + 28 day study visit.

Cases will be those which meet study endpoint definitions as detailed below and occur more than 7 days after their boost + 28 day study visit in the Two Dose for Correlates population. Participants with COVID-19 infection prior to 7 days after their boost + 28 day study visit will be excluded.

The control cohort consists of participants in the Two Dose for Correlates population who have no evidence of infection with COVID-19 up to the data cut-off date for analysis, and for whom immune marker data is available for analysis.

1.2 Study Endpoints

1. Primary symptomatic COVID-19
2. Asymptomatic infection or infection with unknown symptoms (COV002 only)
3. Moderate – severe symptomatic COVID-19 (World Health Organization [WHO] score >= 3.0)
4. Hospitalisation (WHO score >=4.0) (if sufficient cases available)
5. Any RT-PCR+

Sensitivity analysis endpoints

1. Primary symptomatic COVID-19 with/without shortness of breath
2. Primary symptomatic COVID-19 with 3 or more/less than 3 symptoms

1.3 Sample Size

For each main endpoint at least 40 cases will be required for analysis.

1.4 Immune Markers

Main analyses will be conducted using the following assays, with additional exploratory analysis conducted where data become available from additional assays.

1. Spike- and receptor binding domain (RBD)-specific IgG response to SARS-CoV-2 by multiplexed immunoassay (PPD)
2. Antibody neutralisation using a lentivirus-based pseudovirus particle expressing the SARS-CoV-2 spike protein (Monogram)
3. Antibody neutralisation using live SARS-CoV-2 (PHE)

Fold change from pre-boost to post-boost time points may also be explored, as well as the ratio of binding to neutralising antibody.

2. ANALYSIS

2.1 Data summaries

Descriptive summaries of all timepoints will be presented as boxplots and tables of summary statistics, overall and by outcome, according to standard conventions as outlined in the MAA SAP Section 7.1.

Cumulative incidence will be presented by tertile of immune response.

2.2 Risk model (Proxy for SARS-CoV-2 Exposure in participants who received control vaccine)

A baseline risk score will be developed on MenACWY control group participants to use in correlates analysis to adjust for potential confounding due to variability in exposure to SARS-CoV-2 in the participants with antibody data. Only data prior to unblinding will be included in the risk model.

Baseline variables for consideration in the risk model include (but not limited to), age in years, baseline comorbidities (at least one of the following: cardiovascular disorder, respiratory disease, or diabetes), body mass index (BMI ≥ 30 kg/m2 at baseline, non-white ethnicity and health care worker status. Where analyses include data from more than one study/country, then study or country will be included in the risk score. Other variables such as sex and geographic region may also be explored as risk predictors.

The risk score will be developed for Single Dose for Correlates and Two Dose for Correlates controls separately using a logistic regression model with the outcome in the model being one of the study endpoints listed in Section 1.2. A separate risk score will be developed for each study endpoint. If the risk score is highly similar across a set of endpoints, then a single risk score may be selected for application to each endpoint in the set.

The risk score will be developed on the MenACWY control group participants and then used to predict risk of exposure in the participants in the AZD1222 vaccine arm on an individual level. The predicted risk of exposure will be included as a covariate in the Correlates of Risk model (Section 2.4).

2.3 Risk model for availability of antibody data (AZD1222 arm)

Antibody data will not be available for all participants in the AZD1222 vaccine arm of the studies. There will be more data on cases than on non-cases in the vaccine arm, and samples selected for analysis are not entirely random. Therefore, estimates of overall absolute risk of infection calculated from these data will be inflated and result in bias to correlates estimates. To account for this a model will be developed to predict the probability of antibody data being available for inclusion in the analysis based on baseline characteristics in the AZD1222 vaccine arm. Variables used in the prediction model will include age group (18-55 years, 56-69 years, 70 years or above), whether the participant is a case or non-case, the type of case (primary symptomatic, non-primary symptomatic, asymptomatic/unknown symptoms), prime-boost interval and dosage (LD/LD, LD/SD, SD/SD). Probabilities will be exported from the model and the inverse probability used to weight the correlates models described in Section 2.4.

2.4 Correlates of Risk

Models will be derived for each endpoint and immune marker separately, and for each country separately. Combined analyses across countries/studies may also be conducted where feasible.

For each endpoint, generalised additive model (GAM) models for binary data (with logit link functions) will be fitted, adjusting for the baseline risk score from Section 2.2, and weighted by the inverse of the probability derived in Section 2.3. Individual level predicted probabilities will be output from each model and plotted against log-transformed antibody responses (Figure 1). Generalised linear models (GLM) will be fitted as a sensitivity analysis.

Figure 1 Example plot of predicted probabilities against antibody values

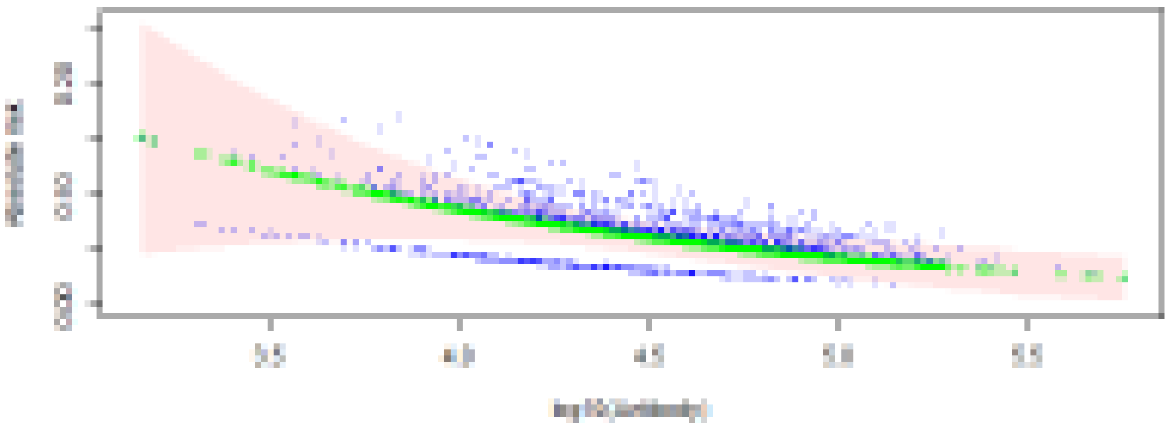

2.5 Correlates of Vaccine Efficacy (CoVE)

To derive the relative risk and correlate of vaccine efficacy, the average risk of infection at each level of antibody response in the vaccine arm, derived from the correlates of risk analysis in Section 2.4, will be divided by the total risk of infection in the MenACWY control group. The total risk in the MenACWY group will be a weighted average of the risk of the outcome across all study groups, weighted by the randomisation ratio in each group. 95% confidence intervals will be derived by bootstrapping.

2.6 Imputation of Censored Data

For some individuals, the antibody titre results are censored as their values are below the lower limit of quantification of the assay (LLOQ). We will impute half the LLOQ for these censored values.

The pseudoneutralising antibody titre contained more censored values (approx. 10%) below the detection limit than the other titres. Brand et al 2019 consider methods for dealing with missing data when a bootstrap is required. They found a single imputation scheme embedded within the bootstrap percentile method outperformed all other methods. As a sensitivity

analysis we will impute the censored pseudoneutralising antibody titre results embedded within the bootstrap, using a Bayesian linear regression or Tobit regression scheme.

3. REFERENCES

Brand et al 2019
Brand J, van Buuren S, le Cessie S, van den Hout W. Combining multiple imputation and bootstrap in the analysis of cost-effectiveness trial data. Statistics in Medicine. 2019; 38: 210–220. https://doi.org/10.1002/sim.7956
