# Supplementary material for: Correlates of protection against symptomatic and asymptomatic SARS-CoV-2 infection
Source: Nat Med. 2021 Sep 29;27(11):2032–40. doi: 10.1038/s41591-021-01540-1 (PMC8604724; doi:10.1038/s41591-021-01540-1)
Supplement: Supplementary file 2 — Reporting Summary [file 41591_2021_1540_MOESM2_ESM.pdf]

nature research

Corresponding author(s): Dr Merryn Voysey

Last updated by author(s): Jun 28, 2021

Reporting Summary

Nature Research wishes to improve the reproducibility of the work that we publish. This form provides structure for consistency and transparency in reporting. For further information on Nature Research policies, see our Editorial Policies and the Editorial Policy Checklist.

Statistics

For all statistical analyses, confirm that the following items are present in the figure legend, table legend, main text, or Methods section.

n/a	Confirmed	
☐	☒	The exact sample size (n) for each experimental group/condition, given as a discrete number and unit of measurement
☐	☒	A statement on whether measurements were taken from distinct samples or whether the same sample was measured repeatedly
☐	☒	The statistical test(s) used AND whether they are one- or two-sided<br>*Only common tests should be described solely by name; describe more complex techniques in the Methods section.*
☐	☒	A description of all covariates tested
☒	☐	A description of any assumptions or corrections, such as tests of normality and adjustment for multiple comparisons
☐	☒	A full description of the statistical parameters including central tendency (e.g. means) or other basic estimates (e.g. regression coefficient) AND variation (e.g. standard deviation) or associated estimates of uncertainty (e.g. confidence intervals)
☐	☒	For null hypothesis testing, the test statistic (e.g. F, t, r) with confidence intervals, effect sizes, degrees of freedom and P value noted<br>*Give P values as exact values whenever suitable.*
☐	☒	For Bayesian analysis, information on the choice of priors and Markov chain Monte Carlo settings
☒	☐	For hierarchical and complex designs, identification of the appropriate level for tests and full reporting of outcomes
☐	☒	Estimates of effect sizes (e.g. Cohen's d, Pearson's r), indicating how they were calculated

Our web collection on statistics for biologists contains articles on many of the points above.

Software and code

Policy information about availability of computer code

Data collection	RedCap 10.6.13 has been used for data collection (© 2021 Vanderbilt University)
Data analysis	Data analysis was done using R version 3.6.1 or later. The GAM was coded using the mgcv package in R. Three knots were used for each GAM, and the smoothing parameter was estimated by generalized cross validation.

For manuscripts utilizing custom algorithms or software that are central to the research but not yet described in published literature, software must be made available to editors and reviewers. We strongly encourage code deposition in a community repository (e.g. GitHub). See the Nature Research guidelines for submitting code & software for further information.

Data

Policy information about availability of data

All manuscripts must include a data availability statement. This statement should provide the following information, where applicable:

- Accession codes, unique identifiers, or web links for publicly available datasets
- A list of figures that have associated raw data
- A description of any restrictions on data availability

Anonymised participant data will be made available when the trials are complete, upon requests directed to the corresponding author. Proposals will be reviewed and approved by the sponsor, investigator, and collaborators on the basis of scientific merit. After approval of a proposal, data can be shared through a secure online platform after signing a data access agreement. All data will be made available for a minimum of 5 years from the end of the trial.

Field-specific reporting

Please select the one below that is the best fit for your research. If you are not sure, read the appropriate sections before making your selection.

☒ Life sciences ☐ Behavioural & social sciences ☐ Ecological, evolutionary & environmental sciences

For a reference copy of the document with all sections, see nature.com/documents/nr-reporting-summary-flat.pdf

Life sciences study design

All studies must disclose on these points even when the disclosure is negative.

Sample size	All available data in the trial have been used in this post-hoc analysis on correlates of protection. The sample size for the trial was based on the primary outcomes which are not reported here.
Data exclusions	Participants were excluded if they were baseline seropositive or unknown to the SARS-CoV-2 N protein at first vaccination, or had their PB28 visit outside a 14 to 42 day window after the second dose, or were followed up to <= 7 days after PB28 with no prior evidence of infection, or received mixed schedules in error (details shown in Figure S1). Exclusions were prespecifed in the statistical analysis plan.
Replication	Replication of findings would be impossible as this is a clinical trial of a vaccine against a rapidly mutating pandemic virus in humans
Randomization	Participants were randomized with full allocation concealment to receive either ChAdOx nCoV-19 vaccine or MenACWY vaccine.
Blinding	This is a single-blinded trial where participants were blinded.

Reporting for specific materials, systems and methods

We require information from authors about some types of materials, experimental systems and methods used in many studies. Here, indicate whether each material, system or method listed is relevant to your study. If you are not sure if a list item applies to your research, read the appropriate section before selecting a response.

Materials & experimental systems

n/a	Involved in the study	
☒	☐	Antibodies
☒	☐	Eukaryotic cell lines
☒	☐	Palaeontology and archaeology
☒	☐	Animals and other organisms
☐	☒	Human research participants
☐	☒	Clinical data
☒	☐	Dual use research of concern

Methods

n/a	Involved in the study	
☒	☐	ChIP-seq
☒	☐	Flow cytometry
☒	☐	MRI-based neuroimaging

Human research participants

Policy information about studies involving human research participants

Population characteristics	UK adults aged 18 years old or above; both male and female; generally healthy
Recruitment	Recruitment was done at 19 sites in the UK with a focus on healthcare workers. No selection biases are known of that would affect results.
Ethics oversight	This study was approved in the UK by the Medicines and Healthcare products Regulatory Agency (MHRA), reference 21584/0428/001 0001, and the South-Central Berkshire Research Ethics Committee, reference 20/SC/0179.<br>All necessary patient/participant informed consent has been obtained and the appropriate institutional forms have been archived.

Note that full information on the approval of the study protocol must also be provided in the manuscript.

Clinical data

Policy information about clinical studies

All manuscripts should comply with the ICMJE guidelines for publication of clinical research and a completed CONSORT checklist must be included with all submissions.

Clinical trial registration	NCT04400838

Study protocol	The study protocol has been appended in previous publications and is accessible through https://doi.org/10.1016/S0140-6736(20)32661-1
Data collection	19 clinical study sites in the England, Scotland and Wales. Participants were enrolled since May 2020 and the study is still ongoing.
Outcomes	Primary symptomatic COVID-19 defined as NAAT+ with at least one of the five COVID symptoms (fever≥ 37.8oC; cough; shortness of breath; anosmia orageusia), and asymptomatic SARS-CoV-2 infections defined as a NAAT+ swab with no symptom reported (see more details in Methods and Statistical Plan in supplementary). Outcomes were predefined in the statistical analysis plan.
